# The interplay between perceived stress, psychological flexibility, and interpretation biases in undergraduate mental health

**DOI:** 10.1038/s41598-025-26492-1

**Published:** 2025-11-07

**Authors:** Alla Machulska, Tim Klucken

**Affiliations:** Department of Clinical Psychology and Psychotherapy, University of Siegen, Obergraben 23, 57072 Siegen, Germany

**Keywords:** Stress, Psychological flexibility, Interpretation bias, Mental health, Depression, Psychology, Human behaviour, Health care

## Abstract

Stress is a well‐established risk factor for psychological burden, yet individual vulnerability varies, suggesting that stress intensity and cognitive-psychological factors might be implicated in this process. This study examined how perceived stress at two different phases (moderate vs. high stress) relates to psychological symptoms and positive mental health through lower levels of psychological flexibility and whether interpretation biases in response to social ambiguity moderate this effect. A total of 228 healthy undergraduate students were assessed once at the beginning of a semester (T0: moderate stress phase) and 12 weeks later during exams (T1: high stress phase). Psychological flexibility mediated the relationship between stress and both psychological symptoms and positive mental health at both time points. Notably, baseline negative interpretation biases moderated this mediation effect for depressive symptoms at T1 (high stress phase), such that lower psychological flexibility was linked to higher depression levels only among individuals with moderate to high levels of negative interpretation bias. In turn, the absence of negative biases buffered against these effects. No such moderation effects were observed under moderate stress (T0) or for positive interpretation biases at either time point. These findings highlight the importance of interventions that combine techniques to foster psychological flexibility and reduce negative interpretation biases, particularly for individuals under high stress or vulnerable to depression.

**Supplementary Information:**

The online version contains supplementary material available at 10.1038/s41598-025-26492-1.

## Introduction

In today’s dynamic and ever-changing world, individuals face a multitude of challenges, which require adequate adaptation to shifting situational demands. Successfully navigating these challenges can foster psychological well-being, whereas difficulties in adapting may lead to psychological distress. In this regard, experiencing continuous stress can be a risk factor for the development of psychological symptoms and emotional disorders (for a recent review, see^1^). Noteworthy, the harmful effects of stress-related responses depend on external conditions such as type, duration, and intensity of a particular stressor^2^ as well as on internal processes^3^. The latter is based on a growing body of evidence indicating that heightened stress can disrupt adaptive emotional regulation and cognitive processing^4,5^. Hence, some individuals might be more vulnerable to the adverse impact of stress and experience stronger negative outcomes on mental health than others ^6,7^. Elucidating major contributing factors to the link between stress and mental health is therefore key as it not only advances scientific understanding but also has practical implications for the development of effective, evidence-based interventions aimed at promoting mental health and reducing the adverse effects of stress.

Recent studies suggest that psychological flexibility plays a crucial mediating role in the relationship between exposure to stress and the development of psychological symptoms as part of stress-related responses^8^ (for reviews, see^9,10^). Psychological flexibility refers to a various set of adaptive abilities, including attending the present moment, thinking with openness, adopting different perspectives, and choosing behavior in accordance with deeply held values^5,11^. Hence, it is reasonable to assume that psychological flexibility can counteract adverse effects following stressful periods in life, thereby maintaining psychological well-being. On the other hand, being exposed to long-lasting stress disrupts adaptive functioning by narrowing cognitive resources and promoting rigid, habitual responses^12,13^, thereby leading to a psychological inflexible state of mind. In line with this notion, several recent studies provide evidence for a bidirectional link between psychological flexibility and stress. For instance, Puolakanaho et al.^14^ assessed adolescents twice during the final year of their basic education and observed concurrent changes in symptoms and psychological flexibility over the school year. Specifically, increases in stress symptoms were accompanied by decreases in psychological flexibility, and vice versa. Moreover, recent cross-sectional and longitudinal research has examined the psychological effects of the Covid-19 pandemic – one of the most significant global stressors in recent history. These studies consistently revealed an association between psychological flexibility and well-being^15,16^ and suggest that psychological flexibility may serve as an important precursor to mental health outcomes^17,18^, especially under conditions of stress.

However, the strength of the relationship between psychological flexibility, and symptoms such as depression, anxiety, and stress has been inconsistent across studies. While some investigations report robust associations^19,20^, others have found non-significant^21^ or only modest effects^22^. One potential explanation for this inconsistency may be that the strength of the association between psychological flexibility and mental health depends on the level of stress. Indeed, under conditions of heightened or chronic stress, more consistent and pronounced associations have been observed^14,23^. Yet, systematic investigations of the flexibility–mental health link across different levels of stress are lacking thus far. Another reason for mixed findings may be that key moderators may not have been fully accounted for in previous research. Indeed, emerging evidence indicates that the impact of stress and/or psychological flexibility on mental health outcomes might be moderated by individual differences in cognitive processes^24^. For instance, according to Lazarus’ seminal model of stress, individuals’ cognitive appraisals play a crucial role in determining whether a given event is perceived as stressful^3^. Expending on this, it is reasonable to assume that cognitive processes might also be implicated in the link between perceived stress and mental health. Moreover, since psychological inflexibility is characterized by rigid responses to different kinds of situations^25^, it is plausible to assume that individuals with a predisposition toward rigid cognitive styles are particularly vulnerable to develop mental health problems. This vulnerability, in turn, may be particularly evident in high-stress contexts, as stress has been implicated in a transition from deliberative to more automatic, habitual response modes ^13^. In this view, those who habitually draw negative conclusions about uncertain or ambiguous events – referred to as negative interpretation bias^26^ – may experience exacerbated mental health symptoms when their psychological flexibility is compromised by stress. Indeed, negative interpretation biases have been reported for depression (for a meta-analysis, see^27^), anxiety^28,29^, and other psychological disorders^30–32^. Moreover, negative interpretation biases have been implicated in the development of stress-related disorders^33^ as well as the susceptibility to stress^34^, indicating that such biases play a crucial role in the onset and maintenance of psychological disorders and mental health problems. To date, however, no study has examined how interpretation biases moderate the relationship between stress, psychological flexibility, and mental health.

Beyond that, although recent advances have promoted a holistic view of mental health that encompasses more than the absence of psychopathology^35^, clinical psychological research still tends to focus more on risk factors and negative outcomes than on protective factors or positive mental health. Considering both negative and positive mental health outcomes aligns well with the Two Continua Model of Mental Health and Illness^36^, which conceptualizes mental health as both the absence of mental illness *and* the presence of mental health. Although different operationalizations exist, most definitions of positive mental health integrate hedonic (i.e., the presence of positive emotions and moods) and eudaimonic (i.e., social functioning) accounts, reflecting general emotional, psychological, and social well-being^37,38^. Assessing positive mental health alongside psychological symptoms thus provides a more comprehensive understanding of individuals’ mental health.

Building on this notion, growing interest has emerged in understanding not only the impact of negative but also positive interpretation biases on mental health (i.e., the tendency to interpret ambiguous situations in a favorable or benign manner^39,40^). Building on these insights, the present study adopts a balanced approach by investigating the impact of both negative *and* positive interpretation biases on both negative (i.e., depression, anxiety, and stress-related symptoms) *and* positive mental health outcomes.

Given that young adulthood is a particularly vulnerable period characterized by high personal and professional demands^41,42^, and that most mental disorders first emerge during adolescence and early adulthood^43,44^, examining mental health during this pivotal phase is essential. The transition into university life adds further stressors, such as heightened demands for self-management, time constraints, and exam pressure, resulting in a higher prevalence of psychological symptoms among students than their working-age peers^45,46^. Therefore, the present study focused on undergraduate students to better understand the interplay of perceived stress and cognitive factors during this formative stage.

We hypothesized that experiencing stress in response to a naturalistic stressor (i.e., going through an academic semester) would be associated with lower psychological flexibility, which in turn would be linked to higher psychological symptoms and lower positive mental health (mediation model). To test this assumption across different levels of stress, healthy undergraduate students were assessed at two different time points: At the beginning of the academic semester (T0) and 12 weeks later at the end of a semester (T1). While both assessment time points can be considered stressful due to increased workload, time constraints, and heightened performance pressure, the end of semester is typically particularly demanding since most exams take place during this time, which can eventually lead to symptom development. Specifically, T1 was scheduled during the first week of the exam period, capturing a period of increased stress and academic demands. This time frame enabled to capture stress effects under both moderate (T0) and high (T1) conditions in an ecologically valid way. Furthermore, we hypothesized that the predisposition to interpret ambiguous social situations in a negative manner (negative interpretation bias) would amplify the adverse relationship between stress and psychological flexibility and/or mental health outcomes as well as the relationship between lower psychological flexibility and mental health (moderated mediation model). Similarly, we expected that positive interpretation biases would mitigate these negative effects on mental health. Overall stronger effects were expected for the high stress period.

## Materials and methods

### Ethics statement

The study protocol was approved by the local Ethics Committee of the University of Siegen and was conducted in accordance with the Declaration of Helsinki and Good Clinical Practice guidelines. Participation was voluntary and participants had the right to withdraw their consent for participation at any time.

### Trial design

The study employed a two-wave design with two measurement time points: An on-site assessment at the beginning at an academic semester (T0), representing a moderate-stress phase, and a 12-weeks online follow-up assessment at the end of a semester (T1), representing a high-stress phase. To examine distinct associations between perceived stress, psychological flexibility, and mental health outcomes (i.e., depression, anxiety, and stress-related symptoms, positive mental health), separate moderated mediation models were conducted for each time point (i.e., one including all variables measured at T0 and one including all variables measured at T1). Building on previous research demonstrating the predictive role of cognitive styles, baseline (T0) positive and negative interpretation biases were included as moderators to examine their influence on the stress–flexibility–mental health outcome relationship.

### Sample size

Sample size was determined based on empirical evidence, methodological recommendations, and practical considerations. Previous research examining the mediating role of psychological flexibility in the relationship between stress and mental health outcomes typically reports medium effect sizes. Building on this, simulation studies indicate that detecting a mediation effect with medium a- and b-paths requires approximately *N* = 78 participants to achieve 80% power when using bias-corrected bootstrap methods^47^. Moderated mediation models generally require larger samples to achieve comparable power. According to Monte Carlo-based simulations, a sample size of at least 200 participants was considered requisite to detect medium effects^48^. While larger samples would be necessary to reliably detect smaller effects, our sampling strategy was informed by feasibility and logistical factors, as data collection occurred in classroom settings as part of an academic course. Based on these empirical benchmarks and practical constraints, the target sample size for the present study was determined to be approximately *N* = 200.

### Participants

A total of 269 participants have been recruited, all of whom attended the same introductory lecture at the University of Siegen, which served as the basis for determining the sample size. Exclusion criteria for all participants were self-reported major medical or psychiatric disorders, insufficient German language skills or uncorrected visual or auditory impairment. Forty-one participants had to be excluded due to these criteria (see Supplemental Material Appendix [SMA], Table A1, for a full description of excluded participants). Therefore, the final sample size comprised *N* = 228. Of those, *n* = 184 self-identified as female, *n* = 42 as male, and *n* = 2 as divers. Participants’ mean age was 22.55 (standard deviation [*SD*]: 4.03; range: 18–48). No information on ethnicity was gathered. Participants received course credit for participation. Fourteen participants failed to complete the second assessment, which indicates a drop-out of 6.1%. Detailed information on participants’ demographic characteristics as well as missing data is provided in the SMA (A2 and A3).

### Procedure

The first assessment (T0) was conducted on-site in a classroom setting and took about 120 min to complete. The second session (T1) was scheduled 12 weeks after the first assessment and was performed during the first week of the exam period. Measures at T1 were carried out online via the Internet platform LimeSurvey.

After providing informed consent, participants completed questions about demographic details, perceived stress, psychological flexibility, and measures of mental health. Thereafter, participants were presented with a measure for positive and negative interpretation biases. Twelve weeks later (T1), participants were asked to complete the same set of questionnaires, which were previously assessed at baseline (T0). As this study was part of a larger study exploring the psychometric properties of different cognitive bias assessment paradigms, participants filled out supplementary questionnaires and performed additional reaction-time based tasks on a personal computer and on a smartphone, which are not the focus of the present trial and therefore will not be presented here. An overview of all measures gathered can be found on https://osf.io/ucf3m/?view_only=ce4dc50ef6ef426b809a309498431793.

### Measures

Measurements relevant to the study’s research questions are presented below in the order in which they have been applied.

#### Perceived stress

Participants were instructed to indicate the extent of perceived stress with respect to the last seven days on a continuum ranging from 0 (not stressed at all) to 100 (extremely stressed).

#### Psychological flexibility

The German version of the Acceptance and Action Questionnaire II (AAQ-II^49^; German: “Fragebogen für Akzeptanz und Handeln [FAH-II]”^50^) was used to assess psychological flexibility defined as the ability to fully contact the present moment, including any aversive internal events^49^. The AAQ-II comprises 10 *inverse* items, which can be rated on 7-point Likert-scales (ranging from 1 = “never true” to 7 = “always true”), resulting in a total score between 10 and 70. Therefore, psychological flexibility is reflected by lower scores on the AAQ-II. According to Bond et al.^49^, scores around 24–28 or higher are associated with elevated symptoms of depression or anxiety. As compared to the first version (AAQ^51^), the AAQ-II has been shown to hold a more stable factor structure and is characterized by a better psychometric test quality^50^. According to established guidelines^52,53^, internal consistency was excellent at both time points (Cronbach’s *α*_*T0*_ = 0.91; *α*_*T1*_ = 0.91).

#### Mental health problems

The German version of the Depression-Anxiety-Stress-Scale-21 (DASS-21^54^; German: DASS-21-G^55^) was used to measure negative emotional states of depression, anxiety, and stress-related symptoms regarding the last seven days. Each scale comprises seven items, which are scored on 4-point Likert-scales (ranging from 0 = “did not apply to me at all” to 3 = “applied to me very much or most of the time”). Higher scores indicate more severe symptoms of psychological distress. Please note that the DASS-21 is not a measure of clinical diagnoses but is conceptualized as a dimensional method to assess psychological burden and mental health problems. Recommended cut-off scores for mild symptom expression are > 4 for the depression subscale, > 3 for the anxiety subscale and > 7 for the stress symptoms subscale^54^. The DASS-21 has been used in different samples (i.e., clinical, and sub-clinical) and age groups and has been shown to be both sensitive to change^56^ and hold good psychometric properties^57^. Internal consistency in this sample was acceptable to good for each subscale and measurement time point (depression subscale: *α*_*T0*_ = 0.88; *α*_*T1*_ = 0.88; anxiety subscale *α*_*T0*_ = 0.77; *α*_*T1*_ = 0.80; stress subscale: *α*_*T0*_ = 0.83; *α*_*T1*_ = 0.84).

#### Positive mental health

The Positive Mental Health Scale (PMH-scale^38^) was used to access positive mental health. The PMH-scale consists of nine general, cross-situational and person-centered items that measure internal factors (e.g., emotional, psychological) of positive mental health. Items are rated on a four-point Likert-scale (ranging from 1 = “not true” to 4 = “true”). For German student samples, a cut-off of 18 for high levels of positive mental health has been recommended^58^. The PMH-scale holds good psychometric properties and can be regarded a brief instrument for measuring psychological well-being and positive mental health across different samples, including students^38^. For this sample, internal consistency was good at both time points (Cronbach’s *α*_*T0*_ = 0.88; *α*_*T1*_ = 0.89).

#### Interpretation bias assessment

Positive and negative interpretation biases were assessed independently using the Ambiguous Social Scenarios Questionnaire (ASSQ^59^), a recently developed adaptation of the Ambiguous Scenarios Questionnaire (ASQ^60^). While both approaches resemble each other in that they provide participants with ambiguous scenarios, the ASSQ defers from the original ASQ in two crucial aspects: First, the ASQ employs a combination of free-response and forced-choice formats to choose between two alternative explanations (threat/nonthreat) for each scenario, thereby requiring subsequent coding and precluding an independent assessment of positive and negative interpretation biases. The ASSQ^59^ was specifically developed to overcome these limitations by providing two explanations (benign/positive vs. threatening/negative), which can be rated independently. Hence, the ASSQ allows for independently assessing positive and negative interpretation biases. Second, the ASQ comprises 12 ambiguous scenarios in total (six social and six body-related scenarios). In contrast, the ASSQ as developed and validated by Baumgardner et al.^59^ includes only the six social scenarios. During the task, participants are instructed to read each scenario carefully and to imagine being in the situation in question (i.e., “You have visitors over for a meal and they leave sooner than expected. Think about why this happened to you.”). Next, a benign/positive and a threatening/negative interpretation for each scenario are provided (i.e., “They had to go somewhere else.” vs. “They were bored and weren’t enjoying themselves.”). Participants are asked to rate how likely they would be to think the two interpretations on 5-point Likert-scales (ranging from 1 = “I would not think that at all” to 5 = “I would immediately think that”). Thus, scores for positive and negative interpretation biases range between 6 and 30, respectively. To prevent sequence effects, the order of benign/positive and threatening/negative interpretations varies across the six scenarios. The ASSQ has been shown to be a time efficient, valid, and reliable measure of positive and negative interpretation biases toward ambiguous social situations^59^. In the present sample, internal consistency for positive interpretation biases was slightly below the conventional threshold of 0.70 (Cronbach’s *α*_*T0*_ = 0.69; *α*_*T1*_ = 0.64), while reliability for negative interpretation biases reached the acceptable range (Cronbach’s *α*_*T0*_ = 0.70; *α*_*T1*_ = 0.71). Test–retest reliability across the 12-week interval was moderate for both biases (Intraclass Correlation Coefficient *ICC*_*positiv*_ = 0.64; *ICC*_*negativ*_ = 0.72), in line with guidelines proposed by Koo and Li^61^.

### Data preparation and statistical analyses

Data were analyzed using IBM SPSS Statistics for Windows, version 29 (IBM Corp.^62^). Loss of information due to missing data was accounted for by Expectation–Maximization (EM) imputation. Given that all missing values occurred systematically at T1, we assumed that data were not missing completely at random (MCAR) but missing at random (MAR^63,64^). Baseline values for each construct of interest (e.g., psychological flexibility, mental health outcomes) were included as predictors in the imputation model to preserve the interrelations among variables. The resulting imputed dataset was subsequently used in all further analyses.

**Fig. 1:**
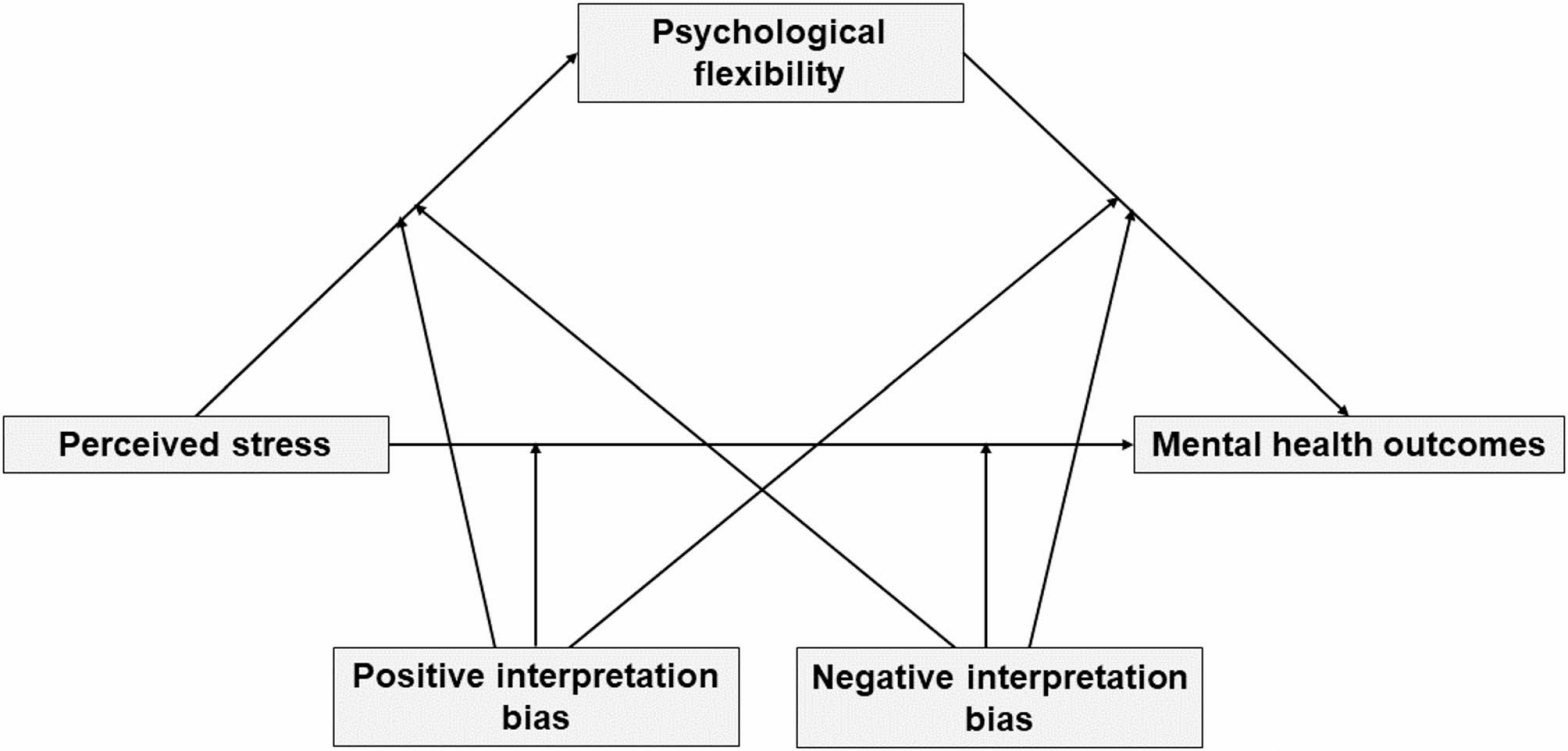
Conceptual diagram.

To examine the mediating role of psychological flexibility in the relationship between perceived stress and mental health under moderate (T0) and high stress (T1) conditions – and to test whether interpretation biases moderate this relationship – we estimated four moderated mediation models for each time point using PROCESS macro Model 76^65^. An illustration for the conceptual moderated mediation model is provided in Fig. 1.

For the moderate-stress condition, all predictors, mediators, and outcomes measured at T0 were included. For the high-stress condition, the same approach was applied with T1 data. Separate models were conducted for each outcome variable (depression, anxiety, stress-related symptoms, and positive mental health). To test the moderating role of interpretation biases, baseline (T0) positive and negative biases were entered as moderators in both the T0 and T1 models. This specification allowed the moderator to influence both the indirect paths (a: stress → psychological flexibility; b: psychological flexibility → outcome) and the direct path (c’: stress → outcome). In line with Hayes^65^, we conducted model pruning when mediators or moderators were not statistically significant, with significant predictors from the initial model being included as covariates to account for their possible effects. Specifically, non-significant interaction terms were removed from the model to simplify interpretation and reduce the risk of overfitting. Detailed results for these models are provided in the SMA A4. All variables included in the models were mean centered to reduce multicollinearity and to enhance the interpretability of the interaction effects.

Prior to examining the predictive power of the regression models, a thorough assessment of underlying assumptions was carried out. Linear relationships between predictors and the dependent variable were examined by means of scatterplots, which revealed no deviations from linear expectations. The Durbin-Watson statistics were near the value of 2 and within the limits of 1.5 and 2.5 for all regression models, indicating that autocorrelation among residuals did not appear, therefore confirming that the assumption of independence of errors was met. The Variance Inflation Factor (*VIF*) for each centered predictor and model was well below the threshold of 10, indicating that multicollinearity did not apply to our data. A diagnostic of influential cases revealed that less than 5% of cases had standardized residuals outside the limits of ± 2.5. Accordingly, all cases were retained for the analyses. Normality assumptions were addressed by employing bootstrap inference for model coefficients. The significance of a moderated mediation or an indirect effect were determined using bootstrapping with 10,000 samples, as suggested by Biesanz et al.^66^. To further ensure robust inference in the presence of heteroscedasticity, heteroscedasticity‐consistent standard errors (HC3^67^) were applied.

## Results

### Participants’ characteristics regarding perceived stress, psychological flexibility, interpretation biases, and mental health outcomes

At baseline, participants reported a moderate level of perceived stress (*M* = 49.53; *SD* = 23.58; observed range: 0–95), which increased at follow-up (*M* = 59.42; *SD* = 21.18; range: 0*–*100; *t*(227) = − 5.58, *p* < 0.001). Participants’ self-reported psychological flexibility was comparable across assessment points (*t*(227) = − 1.00;* p* = 0.320) and its mean did not exceed the cut-off of ≥ 24 (*M*_*T0*_ = 20.20, *SD* = 9.43; *M*_*T1*_ = 20.63, *SD* = 8.73; range at both time points: 7–46). Participants’ endorsements of positive interpretations (*M* = 19.69; *SD* = 3.97; range: 7–29) was higher than those of negative interpretations (*M* = 17.30; *SD* = 3.31; range: 6–29). A paired sample t-test confirmed that this difference was statistically significant (*t*(227) = 4.87; *p* < 0.001).

For both time points, self-reported depression (*M*_*T0*_ = 4.78, *SD* = 4.20; range_T0_: 0–20; *M*_*T1*_ = 5.04, *SD* = 4.14; range_T0_: 0–18; *t*(227) = − 1.25; *p* = 0.213) and anxiety symptoms (*M*_*T0*_ = 3.77, *SD* = 3.60; range_T0_: 0–15; *M*_*T1*_ = 3.73, *SD* = 3.45; range_T0_: 0–14; *t*(227) = 0.23; *p* = 0.817) were slightly above the cut-off for mild symptom occurrence. Stress-related symptoms were beneath the recommended cut-off of > 7 (*M*_*T0*_ = 6.41, *SD* = 4.06; range_T0_: 0–18; *M*_*T1*_ = 6.89, *SD* = 4.20; range_T0_: 0–18) and increased significantly from baseline to follow-up (*t*(227) = − 2.06; *p* < 0.041). Participants’ positive mental health scores exceeded the recommended cut-off of 18 for both assessment times (*M*_*T0*_ = 18.45, *SD* = 5.10; *M*_*T1*_ = 18.36, *SD* = 5.10; range for both time points: 4–27) and remained stable across assessment times (*t*(227) = 0.40; *p* = 0.691).

### Correlations between perceived stress, psychological flexibility, interpretation biases, and mental health outcomes

Correlations between perceived stress, psychological flexibility, interpretation biases, and mental health outcomes (i.e., depression, anxiety, stress, and positive mental health) for the moderate-stress phase (T0) can be found in Table 1.

**Table 1:** Correlations between perceived stress, psychological flexibility, interpretation biases, and mental health outcomes at T0 (moderate stress phase).

	1st Assessment (T0)						
	1	2	3	4	5	6	7
1. Perceived stress	–						
2. Baseline Positive IB	− .002	–					
3. Baseline Negative IB	− .038	− .603***	–				
4. PF	.135*	− .351***	.419***	–			
5. Depression symptoms	.106	− .222***	.325***	.670***	–		
6. Anxiety symptoms	.235***	− .266***	.275***	.639***	.573***	–	
7. Stress-related symptoms	.275***	− .266***	.360***	.647***	.690***	.671***	–
8. PMH	− .162*	.410***	− .349***	− .726***	− .668***	− .523***	− .604***

IB = Interpretation bias; PF = psychological flexibility as assessed with the AAQ-II^49^, which is based on inverse items. Hence, low levels are indicative of psychological flexibility; PMH = positive mental health. *p < .05, **p < .01, ***p < .001.

Perceived stress was neither correlated to positive nor to negative interpretation biases (*rs* ≥ − 0.038; *ps* ≥ 0.566), but it was significantly correlated to psychological flexibility (*r* = 0.135; *p* = 0.041), anxiety (*r* = 0.235; *p* < 0.001), stress-related symptoms (*r* = 0.275; *p* < 0.001), and positive mental health (*r* = − 0.162; *p* = 0.014). Positive and negative interpretation biases correlated significantly with psychological flexibility (*r*_*positive*_ = − 0.351, *p* < 0.001; *r*_*negative*_ = 0.419; *p* < 0.001), psychological symptoms (*rs*_*positive*_ ≤ − 0.222, *ps* < 0.001; *rs*_*negative*_ ≥ 0.275; *ps* < 0.001), and positive mental health (*r*_*positive*_ = 0.410, *p* < 0.001; *r*_*negative*_ = − 0.349; *p* < 0.001). As expected, strong correlations appeared between psychological flexibility and psychological symptoms (*rs* ≥ 0.639; *ps* < 0.001) as well as positive mental health (*r* = − 0.726; *p* < 0.001).

Table 2 shows correlations between perceived stress, psychological flexibility, interpretation biases, and mental health outcomes for the high-stress phase (T1).

**Table 2:** Correlations between perceived stress, psychological flexibility, interpretation biases, and mental health outcomes at T1 (high stress phase).

	2nd Assessment (T1)						
	1	2	3	4	5	6	7
1. Perceived stress	–						
2. Baseline Positive IB	− .122	–					
3. Baseline Negative IB	.141*	− .603***	–				
4. PF	.251***	− .349***	.403***	–			
5. Depression symptoms	.195**	− .293***	.412***	.688***	–		
6. Anxiety symptoms	.187**	− .281***	.343***	.626***	.633***	–	
7. Stress-related symptoms	.297**	− .337***	.396***	.670***	.702***	.719***	–
8. PMH	− .208**	.347***	− .321***	− .663***	− .656***	− .489***	− .567***

IB = Interpretation bias; PF = psychological flexibility as assessed with the AAQ-II^49^, which is based on inverse items. Hence, low levels are indicative of psychological flexibility; PMH = positive mental health. *p < .05, **p < .01, ***p < .001.

Overall, the pattern of associations was largely comparable across time points. Notable exceptions emerged for the significant (but small) correlations between negative interpretation biases and perceived stress at T1 (*r* = 0.141; *p* = 0.034) and between perceived stress and depression symptoms at T1 (*r* = 0.195; *p* = 0.003). These correlations were not significant at T0. In addition, the association between perceived stress and psychological flexibility was more pronounced at T1 (*r* = 0.251; *p* < 0.001) than at T0 (*r* = 0.135; *p* = 0.041). These findings provide preliminary indication that the links between stress and related variables become more pronounced under heightened stress conditions.

### Moderated mediation for the moderate stress phase (T0)

The models assessed the mediating role of psychological flexibility in the relationship between perceived stress and mental health outcomes (depression, anxiety, stress-related symptoms, positive mental health) during the moderate stress period (T0). Baseline positive and negative interpretation biases were entered jointly as moderators. As the a-path (perceived stress → psychological flexibility, with interpretation biases serving as the moderators) applied to all mental health outcomes, results of these models will be reported first.

Detailed results can be found in Table 3.

**Table 3:** Regression results for the a-path at T0 (moderate stress phase).

Variable	Model a-path		
	*b*	SE	*p*
Perceived stress (X)	**.059**	**.025**	**.018**
Negative interpretation bias (W)	**.730**	**.174**	** < .001**
Positive interpretation bias (Z)	− .360	.203	.078
Interaction 1 X*W	.013	.007	.068
Interaction 2 X*Z	.011	.008	.189
Explained variance *R*^2^	**.228**		** < .001**

Significant results are presented in bold.

As can be seen, neither the positive (*b* = 0.011, *p* = 0.189, Δ*R*^2^ = 0.009, bootstrapped *CI* [− 0.006, 0.026]) nor the negative interpretation bias (*b* = 0.013, *p* = 0.067, Δ*R*^2^ = 0.015, bootstrapped *CI* [− 0.002, 0.025]) moderated the path between perceived stress and psychological flexibility at T0.

#### Depression symptoms

Both interpretation biases did not significantly moderate the b- (for negative biases: *b* = 0.005, *p* = 0.524, Δ*R*^2^ = 0.001, bootstrapped *CI* [− 0.008, 0.019]); for positive biases: *b* = − 0.002, *p* = 0.832, Δ*R*^2^ < 0.001, bootstrapped *CI* [− 0.017, 0.015]) or c’-path (for negative biases:* b* = − 0.003, *p* = 0.191, Δ*R*^2^ = 0.005, bootstrapped *CI* [− 0.009, 0.002]); for positive biases: *b* = 0.001, *p* = 0.771, Δ*R*^2^ < 0.001, bootstrapped *CI* [− 0.004, 0.006]). Complete regression results can be found in Table 4.

**Table 4:** Regression results for the b- and c’-path at T0 (moderate stress phase).

Model b/c ‘-path	Mental health outcomes											
	Depression symptoms			Anxiety symptoms			Stress symptoms			Positive mental health		
Variables	*b*	*SE*	*p*	*b*	*SE*	*p*	*b*	*SE*	*p*	*b*	*SE*	*p*
Perceived stress (X)	.005	.011	.617	**.023**	**.008**	**.003**	**.036**	**.101**	** < .001**	− .017	.011	.126
Psychological flexibility (M)	**.288**	**.030**	** < .001**	**.228**	**.024**	** < .001**	**.239**	**.029**	** < .001**	− **.376**	**.039**	** < .001**
Negative interpretation bias (W)	.091	.056	.109	− .002	.057	.968	.139	.076	.069	.072	.067	.280
Positive interpretation bias (Z)	.064	.060	.290	− .046	.064	.472	.019	.077	.808	**.255**	**.094**	**.007**
Interaction 1 M*W	.005	.007	.524	.001	.006	.845	.005	.008	.513	**.018**	**.009**	**.045**
Interaction 2 M*Z	− .002	.008	.832	− .006	.007	.368	− .001	.009	.891	.011	.014	.458
Interaction 3 X*W	− .003	.003	.191	− .001	.002	.450	− .001	.003	.729	.002	.003	.538
Interaction 4 X*Z	.001	.003	.771	− .002	.002	.349	.001	.003	.717	.002	.004	.571
Explained variance *R*^2^	**.446**		** < .001**	**.441**		** < .001**	**.475**		** < .001**	**.578**		** < .001**

Significant results are presented in bold.

Given that interpretation biases did not emerge as significant moderators for any model paths, process model 4 (mediation-only model) was employed to examine whether psychological flexibility was a significant mediator for the association between perceived stress and depression symptoms. Results revealed a significant indirect effect of stress on depression through lower levels of flexibility (*b* = 0.018, bootstrapped *CI* [0.004, 0.033]), providing evidence for a mediation.

#### Anxiety symptoms

Both interpretation biases did not significantly moderate the b- (for negative biases:* b* = 0.001, *p* = 0.845, Δ*R*^2^ < 0.001, bootstrapped *CI* [− 0.010, 0.013]); for positive biases: *b* = − 0.006, *p* = 0.368, Δ*R*^2^ = 0.002, bootstrapped *CI* [− 0.018, 0.007]) or c’-path (for negative biases:* b* = − 0.001, *p* = 0.450, Δ*R*^2^ = 0.001, bootstrapped *CI* [− 0.005, 0.003]); for positive biases: *b* = − 0.002, *p* = 0.349, Δ*R*^2^ = 0.002, bootstrapped *CI* [− 0.006, 0.003]). Complete regression results can be found in Table 4.

Process model 4 was used to examine whether psychological flexibility was a significant mediator for the association between perceived stress and anxiety symptoms. Results revealed a significant indirect effect of stress on anxiety through lower levels of flexibility (*b* = 0.014, bootstrapped *CI* [0.003, 0.026]), providing evidence for a mediation effect.

#### Stress-related symptoms

Both interpretation biases did not significantly moderate the b- (for negative biases:* b* = 0.005, *p* = 0.513, Δ*R*^2^ = 0.002, bootstrapped *CI* [− 0.009, 0.020]); for positive biases: *b* = − 0.001, *p* = 0.891, Δ*R*^2^ < 0.001, bootstrapped *CI* [− 0.016, 0.015]) or c’-path (for negative biases:* b* = − 0.001, *p* = 0.729, Δ*R*^2^ < 0.001, bootstrapped *CI* [− 0.007, 0.005]); for positive biases: *b* = 0.001, *p* = 0.717, Δ*R*^2^ < 0.001, bootstrapped *CI* [− 0.005, 0.008]). Complete regression results can be found in Table 4.

Process model 4 was used to examine whether psychological flexibility was a significant mediator for the association between perceived stress and stress-related symptoms. Results revealed a significant indirect effect of stress on stress-related symptoms through lower levels of flexibility (*b* = 0.016, bootstrapped *CI* [0.003, 0.030]), providing evidence for a mediation effect.

#### Positive mental health

Regarding the b-path, negative interpretation biases moderated the link between psychological flexibility and positive mental health (*b* = 0.018, *p* = 0.045, Δ*R*^2^ = 0.012, bootstrapped *CI* [0.0002, 0.033]). No other interactions were significant (for the b-path and positive biases: *b* = 0.011, *p* = 0.458, Δ*R*^2^ = 0.004, bootstrapped *CI* [− 0.013, 0.034]; for the c’-path and negative biases:* b* = 0.002, *p* = 0.538, Δ*R*^2^ < 0.001, bootstrapped *CI* [− 0.003, 0.008]); for the c’-path and positive biases: *b* = 0.002, *p* = 0.571, Δ*R*^2^ = 0.001, bootstrapped *CI* [− 0.005, 0.010]). Complete regression results can be found in Table 4.

Given the significant interaction between psychological flexibility and negative interpretation biases, process model 14 (moderated mediation with a moderated b-path) was used for further examination. Contrary to the results above, the index of moderated mediation (*b* = 0.001) was not significant as the bootstrapped CI included zero (− 0.0001, 0.002). Thus, negative interpretation biases did not significantly moderate the b-path in this model. As with mental health symptoms, results did only provide support for a mediation effect of perceived stress on positive mental health through lower psychological flexibility, as evidenced by the significant indirect effect in model 4 (*b* = − 0.021, bootstrapped *CI* [− 0.038, − 0.004]).

### Moderated mediation for the high stress phase (T1)

The models assessed the mediating role of psychological flexibility in the relationship between perceived stress and mental health outcomes (depression, anxiety, stress-related symptoms, positive mental health) during the high stress period (T1). Baseline positive and negative interpretation biases were entered jointly as moderators. Similarly to the models for T0, the a-path applied to all mental health outcomes, which is why results of these models will be reported first.

Detailed results can be found in Table 5. As can be seen, neither the positive (*b* = − 0.006, *p* = 0.557, Δ*R*^2^ = 0.002, bootstrapped *CI* − 0.024, 0.014]) nor the negative interpretation bias (*b* = − 0.009, *p* = 0.316, Δ*R*^2^ = 0.005, bootstrapped *CI* [− 0.021, 0.011]) moderated the path between perceived stress and psychological flexibility at T1.

**Table 5:** Regression results for the a-path at T1 (high stress phase).

Variable	Model a-path		
	*b*	*SE*	*p*
Perceived stress (X)	**.084**	**.030**	**.005**
Negative interpretation bias (W)	**.587**	**.165**	** < .001**
Positive interpretation bias (Z)	− .322	.183	.080
Interaction 1 X*W	− .009	.009	.316
Interaction 2 X*Z	− .006	.011	.557
Explained variance *R*^2^	**.022**		** < .001**

Significant results are presented in bold.

#### Depression symptoms

Regarding the b-path, negative interpretation biases moderated the link between psychological flexibility and depression symptoms (*b* = 0.020, *p* = 0.001, Δ*R*^2^ = 0.023, bootstrapped *CI* [0.008, 0.032]). No other interactions were significant (for the b-path and positive biases: *b* = 0.010, *p* = 0.164, Δ*R*^2^ = 0.004, bootstrapped *CI* [− 0.005, 0.024]; for the c’-path and negative biases:* b* = 0.002, *p* = 0.430, Δ*R*^2^ = 0.001, bootstrapped *CI* [− 0.004, 0.007]); for the c’-path and positive biases: *b* = 0.001, *p* = 0.828, Δ*R*^2^ < 0.001, bootstrapped *CI* [− 0.006, 0.006]). Complete regression results can be found in Table 6.

**Table 6:** Regression results for the b- and c’-path at T1 (high stress phase).

Model b/c ‘-path	Mental health outcomes											
	Depression symptoms			Anxiety symptoms			Stress symptoms			Positive mental health		
Variables	*b*	*SE*	*p*	*b*	*SE*	*p*	*b*	*SE*	*p*	*b*	*SE*	*p*
Perceived stress (X)	.005	.011	.661	.005	.010	.539	**.027**	**.010**	**.009**	− .008	.014	.577
Psychological flexibility (M)	**.283**	**.030**	** < .001**	**.227**	**.025**	** < .001**	**.269**	**.030**	** < .001**	− **.365**	**.045**	** < .001**
Negative interpretation bias (W)	**.181**	**.057**	**.002**	.079	.053	.135	**.121**	**.061**	**.048**	.029	.073	.698
Positive interpretation bias (Z)	.041	.057	.472	− .015	.060	.802	− .048	.062	.438	**.187**	**.085**	**.030**
Interaction 1 M*W	**.020**	**.006**	**.001**	.004	.005	.494	.011	.006	.091	.011	.008	.177
Interaction 2 M*Z	.010	.007	.164	.005	.007	.463	.010	.007	.166	.010	.011	.338
Interaction 3 X*W	.002	.003	.430	.002	.002	.392	.001	.003	.764	-.002	.003	.618
Interaction 4 X*Z	.001	.003	.828	− .002	.003	.602	< − .001	.003	.916	− .001	.004	.750
Explained variance *R*^2^	**.523**		** < .001**	**.411**		** < .001**	**.494**		** < .001**	**.462**		** < .001**

Significant results are presented in bold.

**Fig. 2:**
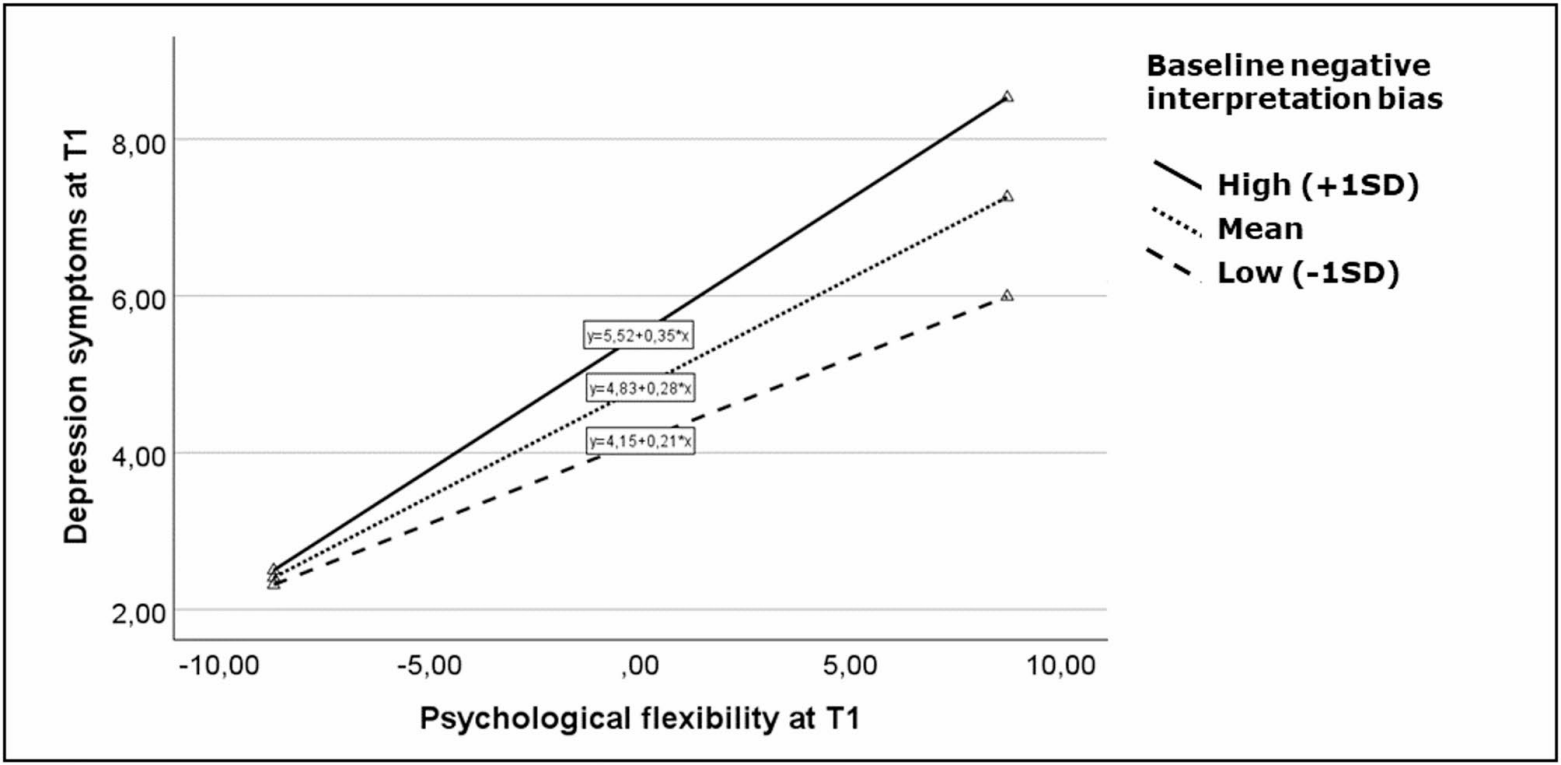
Association between psychological flexibility on depression symptoms in the high stress phase (T1) at low, medium, and high baseline values of negative interpretation bias. Variables are mean-centered. Psychological flexibility was measured with the Acceptance and Action Questionnaire II (AAQ-II ^49^), where low values represent high psychological flexibility.

Given the significant interaction between psychological flexibility and negative interpretation biases, process model 14 was used for further examination. The index of moderated mediation was significant, providing evidence for a moderating role of negative interpretation biases on the psychological flexibility-depression link at T1 (*b* = 0.001, bootstrapped *CI* [0.0003, 0.003]). Follow-up analyses revealed that the effect of reduced psychological flexibility on depression scores was strongest for high negative interpretation bias values (*b* = 0.346, *p* < 0.001, 95% percentile *CI* [0.290, 0.402]), it was lower for medium interpretation biases (*b* = 0.279, *p* < 0.001, 95% percentile *CI* [0.224, 0.333]), and lowest (but still significant) for low interpretation biases (*b* = 0.211, *p* < 0.001, 95% percentile *CI* [0.126, 0.296]). Based on the Johnson-Neyman method, results indicated that for negative interpretation biases below ≤ –2.6 *SD*, the conditional effect of psychological flexibility on depression was no longer significant. Moderated effects for the b-path are shown in Fig. 2.

**Fig. 3:**
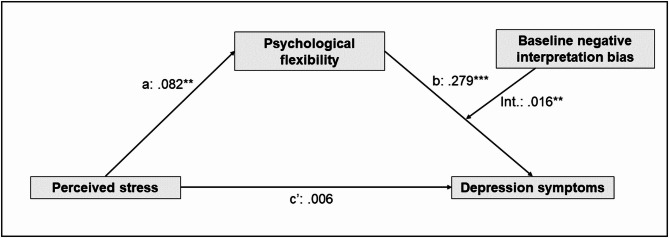
Moderated mediation model for the link between perceived stress on depression through psychological flexibility in the high stress phase, moderated by baseline negative interpretation biases*.* Results are based on process model 14 (moderated mediation model with a moderated b-path); *p < .05, **p < .01, ***p < .001.

The complete moderated mediation model for depression symptoms is presented in Fig. 3.

#### Anxiety symptoms

Both interpretation biases did not significantly moderate the b- (for negative biases:* b* = 0.004, *p* = 0.494, Δ*R*^2^ = 0.001, bootstrapped *CI* [− 0.007, 0.015]); for positive biases: *b* = 0.005, *p* = 0.463, Δ*R*^2^ = 0.001, bootstrapped *CI* [− 0.007, 0.018]) or c’-path (for negative biases:* b* = 0.002, *p* = 0.392, Δ*R*^2^ = 0.002, bootstrapped *CI* [− 0.003, 0.007]); for positive biases: *b* = − 0.002, *p* = 0.602, Δ*R*^2^ = 0.001, bootstrapped *CI* [− 0.008, 0.005]). Complete regression results can be found in Table 6.

Process model 4 was used to examine whether psychological flexibility was a significant mediator for the association between perceived stress and anxiety symptoms. Results revealed a significant indirect effect of stress on anxiety through lower levels of flexibility (*b* = 0.020, bootstrapped *CI* [0.007, 0.034]), providing evidence for a mediation effect.

#### Stress-related symptoms

Both interpretation biases did not significantly moderate the b- (for negative biases:* b* = 0.011, *p* = 0.091, Δ*R*^2^ = 0.007, bootstrapped *CI* [− 0.002, 0.032]); for positive biases: *b* = 0.010, *p* = 0.166, Δ*R*^2^ = 0.004, bootstrapped *CI* [− 0.005, 0.023]) or c’-path (for negative biases:* b* = 0.001, *p* = 0.764, Δ*R*^2^ < 0.001, bootstrapped *CI* [− 0.004, 0.008]); for positive biases: *b* < − 0.001, *p* = 0.416, Δ*R*^2^ < 0.001, bootstrapped *CI* [− 0.005, 0.007]). Complete regression results can be found in Table 6.

Process model 4 was used to examine whether psychological flexibility was a significant mediator for the association between perceived stress and stress-related symptoms. Results revealed a significant indirect effect of stress on stress-related symptoms through lower levels of flexibility (*b* = 0.023, bootstrapped *CI* [0.008, 0.039]), providing evidence for a mediation effect.

#### Positive mental health

Both interpretation biases did not significantly moderate the b- (for negative biases:* b* = 0.011, *p* = 0.177, Δ*R*^2^ = 0.005, bootstrapped *CI* [− 0.005, 0.027]); for positive biases: *b* = 0.010, *p* = 0.338, Δv = 0.003, bootstrapped *CI* [− 0.010, 0.030]) or c’-path (for negative biases:* b* = − 0.002, *p* = 0.618, Δ*R*^2^ = 0.001, bootstrapped *CI* [− 0.008, 0.005]); for positive biases: *b* < − 0.001, *p* = 0.750, Δ*R*^2^ < 0.001, bootstrapped *CI* [− 0.010, 0.007]). Complete regression results can be found in Table 6.

Process model 4 was used to examine whether psychological flexibility was a significant mediator for the association between perceived stress and positive mental health. Results revealed a significant indirect effect of stress on mental health through lower levels of flexibility (*b* = − 0.029, bootstrapped *CI* [− 0.050, 0.010]), providing evidence for a mediation effect.

## Discussion

The study investigated the link between perceived stress and psychological flexibility and how this interacts with interpretative biases to influence mental health outcomes of undergraduate students. To achieve this, participants were assessed at two distinct time points: Once at the beginning (T0) and once at the end (T1) of an academic semester. While perceived stress was moderate at the first assessment (moderate stress phase), the second assessment was placed within the exam period, leading to additional increases in participants’ perceived stress and stress-related symptoms (high stress phase). Comparing these two periods may offer valuable insights into crucial psychological factors that influence mental health during varying levels of demand. We hypothesized that experiencing stress would negatively impact psychological flexibility and that this in turn would influence participants’ mental health – reflected in psychological symptoms and positive mental health – over and above direct effects of stress. In addition, we expected positive and negative interpretation biases to moderate the link between stress, psychological flexibility, and mental health. Specifically, we examined whether these effects would be more pronounced under high-stress conditions (T1) as compared to moderate-stress conditions (T0).

Overall, moderate-sized correlations were observed between stress, psychological flexibility, interpretation biases, and mental health outcomes (i.e., depression, anxiety, stress-related symptoms, and positive mental health). This pattern of associations was largely consistent across both time points. However, some associations emerged only under high-stress conditions (i.e., between negative interpretation biases and perceived stress or between perceived stress and depression symptoms). In addition, the association between perceived stress and psychological flexibility was stronger at T1 than at T0. These findings provide preliminary evidence that differentiating levels of stress may be meaningful, as the high-stress condition appeared to amplify certain associations between cognitive styles, stress, and mental health symptoms that were less pronounced under moderate stress. This may also help explain some of the inconsistencies reported in the literature^21,22^, suggesting that the degree of stress exposure could be an important factor to consider in future research.

Our findings confirm psychological flexibility as a mediator between perceived stress and mental health outcomes at both time points, supporting previous research^16,18^. This aligns with the notion that stress can impair the ability to regulate and accept distressing thoughts and emotions, which in turn can constitute a risk factor for developing maladaptive symptoms or forfeit positive mental health^5^. While stress is a common experience, individual responses vary widely^6,7^. The present study was especially well-suited to examine this, as all participants faced the same academic stressors (T0: preparing for an academic semester; T1: mastering the exam period) yet showed differences in stress-related mental health outcomes.

Moreover, the current trial provides the first evidence that negative interpretation biases moderate the relationship between psychological flexibility and depressive symptoms under high-stress conditions. Low psychological flexibility at T1 was associated with increased depression scores only in participants who exhibited at least mild negative interpretation biases. In the absence of negative interpretation biases, low scores in psychological flexibility had no such negative link with depression. Conversely, for individuals remaining high levels of psychological flexibility under conditions of increased stress, depressive symptoms remained unaffected even at the presence of strong negative interpretation biases, indicating that psychological flexibility can buffer the effects of negative thinking styles. Noteworthy, this moderation was not observed in the moderate stress condition. Hence, our results are in line with recent research highlighting the role of interpretation biases in explaining individual differences in stress responses. For example, in an experimental study, Gonzalez et al.^68^ found that students undergoing a social stress test exhibited greater autonomic reactivity and poorer recovery rates when they had threat-related interpretation biases, as measured by the Word-Sentence Association Paradigm for Youth and Young Adults (WSAP-Y^69^). Likewise, Feng et al.^70^ adopted a multi-wave cross-sectional design and showed that interpretation biases contributed to changes in psychological symptoms (i.e., worry and anxiety) during future stressful contexts. This suggests that interpretation biases may be implicated in the mechanisms underlying stress effects. The finding that the moderating effect of negative interpretation biases was only observed at T1 (a period of high stress) suggests that such cognitive biases may function as latent vulnerabilities that become activated primarily under conditions of elevated demands and strain. In these situations, they may interact with key psychological processes, such as psychological flexibility, thereby contributing to adverse outcomes. Our findings are consistent with theoretical perspectives and empirical evidence indicating a robust link between stressful life events and the onset of mental disorders, particularly depressive episodes (for a review, see ^71^). Moreover, the present study underscores the importance of moving beyond a unidirectional stress–depression framework toward a more dynamic perspective that considers the interplay between stress and individual characteristics such as psychological flexibility and cognitive biases. If replicated, our study extends previous findings by examining effects of distinct naturalistic stressors of varying levels of intensity and providing valuable insights into how cognitive biases relate to sustained stress responses in real-world settings.

In particular, the present trial provides insights into the psychological working mechanisms between stress, psychological flexibility, and automatic cognitive processes in contributing to depressive symptoms. It can be argued that the combination of low psychological flexibility *and* the presence of negative interpretation biases acts in the sense of a psychopathological exacerbator, possibly by undermining the capability to deal adaptively with stress^72^. In line with this notion, Everaert et al.^24^ showed that negative interpretation biases were related to repetitive negative thinking in a sample with various depression levels. In addition, inflexible negative interpretations were related to a dampening of positive emotions, which in turn mediated the relation between inflexible interpretations and psychological symptoms. Another study^8^ demonstrated that psychological flexibility was capable of buffering both the impact of major life events *and* the negative appraisal of such events on depressive symptoms. The current trial thus confirms and broadens these results by providing evidence for a specific interplay between psychological flexibility and negative interpretation biases on depression symptoms. By doing so, it points to the need to consider automatic thinking styles (i.e., as expressed in interpretation biases) as a potential moderator.

In addition, as baseline interpretation biases served as moderators in the second (high stress) assessment, our findings may expand previous correlational approaches^24,73,74^. Several longitudinal studies comply with the present work^75,76^, pointing to the fact that interpretation biases are not only a correlate of psychopathological symptoms and well-being, but should be rather regarded as their antecedent.

Although psychological flexibility and (positive and negative) interpretation biases were at least moderately correlated with mental health outcomes, our findings indicated that the interaction between psychological flexibility and negative interpretation biases was significant only for depressive symptoms at T1, with no comparable moderating effects observed for anxiety and stress-related symptoms, or for positive biases and positive mental health. Possible explanations for these null findings may lie in the assessment methods used to measure interpretation biases and/or positive mental health. For instance, the ASSQ^59^, which was employed to assess interpretation biases, may be particularly sensitive to cognitive processes underlying depression. The ambiguous scenarios in the ASSQ primarily depict social situations such as an appointment with a supervisor at short notice or ambiguous behavior among friends, which are likely to be interpreted in a manner that threatens self-esteem and evokes self-doubt, core features of depressive symptomatology. Noteworthy, the ASQ^60^, on which the ASSQ is based, also includes body-related scenarios (i.e., experiencing an increased heart rate) in addition to social situations. It might be that body-related interpretation biases are stronger moderators for anxiety and stress-related symptoms. Extending the ASSQ to incorporate ambiguous body-related scenarios with independent ratings for benign/positive and threatening/negative interpretations could provide further insight into the broader moderating role of interpretation biases across different mental health outcomes. On the other hand, the absence of significant predictive or moderating associations between positive interpretation biases and positive mental health outcomes may be attributable to the characteristics of the measurement tool used for the outcome. The PMH-Scale^38^ conceptualizes positive mental health as a relatively stable trait, which may limit its sensitivity to short-term fluctuations (i.e., 12-week follow-up as in this study). It is possible that the expected relationships would be more detectable using a measure that captures dynamic changes over time, such as the Mental Health Continuum-Short Form (MHC-SF^77^). Indeed, studies employing the MHC-SF have provided at least some evidence that cognitive reappraisal abilities – potentially linked to interpretation biases – play a prognostic role in fostering positive mental health^18^.

Results of the present study can inform the development of individualized intervention programs that focus on the observed mediators and moderators of the stress-mental health relationship. For instance, some endeavors have been made in recent years to experimentally modify maladaptive interpretation biases, known as Cognitive-Bias-Modification for Interpretations (CBM-I; for a review, see^78^). CBM-I is based on the notion that negative interpretation biases can be changed by training participants to resolve ambiguous cues in a rather benign way^79^. Indeed, several studies have shown that CBM-I is capable of both reducing maladaptive biases (i.e., ^80^) as well as providing therapeutic benefits for individuals suffering from mental health issues, including depression^79^. However, positive results were less encouraging for subclinical samples ^81^. The present study provides a possible explanation for these mixed results as it suggests that negative interpretations do not always have to exert detrimental effects on mental health. Indeed, for individuals scoring high on psychological flexibility, this negative effect might be counterbalanced. Conversely, those exhibiting both low psychological flexibility *and* high negative interpretation biases might have a particular vulnerability to the development of psychological symptoms and/or disorders. Hence, combining CBM-I with techniques based on increasing psychological flexibility might have merit for such cases. The Acceptance and Commitment Therapy (ACT; ^72^) intends to contribute to a more flexible and less avoidant dealing with aversive thoughts, emotions, and bodily sensations and has been shown to increase psychological flexibility^11^, especially in individuals reporting elevated levels of work-related stress^82^. Hence, integrating CBM-I with ACT-based techniques may hold promise in clinical and subclinical settings where individuals experience high levels of stress.

While the study exhibits notable strengths, several limitations must also be acknowledged. First, the sample consisted of healthy young adults, most of whom self-identified as female. Hence, results might be not attributable to more diverse populations such as male, non-binary or older participants. Indeed, research suggests age-specific changes in interpretation biases, with older individuals showing stronger positive interpretation biases^83^. This may also help to explain why we did not find positive interpretation biases to be a significant predictor or moderator of mental health. In addition, the generalizability of our findings to clinical populations remains uncertain. However, with regards to interpretation biases, some evidence points to the fact that such biases are partly independent from symptom severity^84^. This suggests that interpretation biases are relevant for both healthy and clinical samples and are promising targets for therapeutic and preventative interventions. In addition, as no information on ethnicity was collected, the generalizability of our findings to different cultural or ethnic groups cannot be determined. Second, the present study employed a broad naturalistic stressor (exam period), which brings the advantage of increasing ecological validity in the context of academic stress. However, stressors encountered in other life circumstances (e.g., full-time work, caregiving responsibilities) may differ substantially from those experienced by undergraduates, making it uncertain whether our findings generalize beyond academic environments. In addition, the lack of experimental control over the stressor might have concealed intermediate or confounding factors. Germane to this, although our decision to include psychological flexibility as a mediator between perceived stress and mental health was grounded in both theoretical frameworks and empirical evidence, our study design renders the investigation of reciprocal relationships between stress and flexibility unfeasible, limiting our ability to draw definitive causal conclusions. Future research employing more fine-grained, time-lagged designs or experimental manipulations will be essential to disentangle the bi-directional influences between stress and psychological flexibility. In addition, while the current sample size was adequate to detect medium-sized indirect and main effects, the study was likely underpowered to detect smaller interaction effects, which are common in moderated mediation models. According to Monte Carlo-based simulation-based guidelines^48^, small conditional effects may require *N* ≥ 400–500. This limitation may partly account for the absence of significant moderation effects. Future research should employ larger samples to ensure sufficient power to detect potential subtle moderated effects. Finally, the ASSQ can be regarded a rather direct assessment tool for interpretation biases^27^. While direct tools offer simple administration and interpretation and allow for the use of more complex stimulus material and response options, they may be influenced by demand characteristics and are less suitable for capturing automatic processing^85^. Given that indirect tasks are not without limitations, particularly regarding reliability issues^86^, integrating both approaches may offer a more comprehensive assessment.

Taken together, the present study demonstrated that psychological flexibility mediated the effects of perceived stress on mental health across both stress conditions (T0: moderate stress; T1: high stress). Specifically, negative interpretation biases amplified the link between low flexibility and depression only during the high stress period, potentially constituting a vulnerability factor. Hence, incorporating interpretation biases into broader cognitive models of mental health as well as treatment and preventative approaches is deemed essential for contributing to improvements in clinical psychological theory and practice. An avenue for future research could be to examine the mechanisms through which interpretation biases interact with psychological flexibility, especially across varying levels of psychological functioning. This allows for a comprehensive understanding of factors that underlie stress responsiveness and determine why some individuals develop maladaptive stress-related symptoms while others remain resilient.

## Supplementary Information

Supplementary Information.

## Data Availability

The dataset used and analyzed during the current study as well as study materials are available at https://osf.io/567ue/?view_only=bb69a18255f145ce9091f3ef6e0701ef.
