# Supplementary material for: The interplay between perceived stress, psychological flexibility, and interpretation biases in undergraduate mental health
Source: Sci Rep. 2025 Nov 7;15:38992. doi: 10.1038/s41598-025-26492-1 (PMC12595107; doi:10.1038/s41598-025-26492-1)
Supplement: Supplementary file 1 — Supplementary Information. [file 41598_2025_26492_MOESM1_ESM.pdf]

Supplemental Material Appendix

This appendix provides additional analyses for participants who were excluded due to not meeting eligibility criteria (A1) and for those who did not complete the second assessment (A2). Furthermore, we report additional participant demographic characteristics (A3) and supplementary results from model pruning procedures (A4).

A1. Overview of self-reported clinical diagnoses among the 41 participants excluded for not meeting eligibility criteria.

Table A1.

Self-reported clinical diagnoses among excluded participants (n=41).

**Self-Reported Diagnosis**	**Number of participants**
Unipolar depression	16
Anxiety disorders	10
Eating disorders	4
Post-traumatic stress disorder (PTSD)	3
Obsessive-Compulsive Disorder (OCD)	3
Attention Deficit Hyperactivity Disorder (ADHS)	3
Personality disorders	3
Other	5

Note. Multiple mentions per participant were possible.

A2. Participant characteristics for those with complete data versus those missing data at T1.

Table A2a.

Participant characteristics for continuous variables.

**Variables**	***missing***	***Mean***	***Standard deviation***	***N***	***F(1,226)***	***p***
Age	0	22,48	3,905	214	1.09	.297
	1	23,64	5,652	14		
Perceived stress	0	49,18	23,598	214	.78	.378
	1	54,93	23,477	14		

Psychological flexibility	,00	19,8131	9,23164	214	**6.05**	**.015**
	1	26,1429	10,83340	14		
Positive interpretation bias	0	19,8832	3,72536	214	**8.24**	**.004**
	1	16,7857	6,20395	14		
Negative interpretation bias	0	17,2757	4,19855	214	.14	.713
	1	17,7143	5,94110	14		
Positive mental health	0	18,6729	4,93769	214	**6.72**	**.010**
	1	15,0714	6,43864	14		
Depression symptoms	0	4,5841	3,98941	214	**7.88**	**.005**
	1	7,7857	6,02787	14		
Anxiety symptoms	0	3,6075	3,50387	214	**7.45**	**.007**
	1	6,2857	4,26846	14		
Stress-related symptoms	0	6,2944	4,00496	214	2.96	.087
	1	8,2143	4,66045	14		

Note. 0 = complete cases; 1 = missing; inference statistics refer to MANOVA analyses.

Table A2b.

Participant characteristics for self-reported gender.

		missing		
		0	1	Sum
Gender	female	173	11	184
	male	39	3	42
	divers	2	0	2
Sum		214	14	228
χ^2 = .212, *p* = .899				

Note. 0 = complete cases; 1 = missing; inference statistics refer to a χ^2-test.

A3. Additional Participant demographic characteristics

	Min	Max	Mean	SD
Age	18	48	22.55	4.027
Number of academic semester	1	15	3.23	1.802

Subject studied	%	Accumulated %
Pre-studies	.9	.9
Psychology	48.2	52.3
Social Work	14.4	67.7
Teaching	30.3	100.0

A4. Supplemental model pruning results

A4a: Moderate stress condition

Depression outcomes (model 4)

```
Model  : 4
    Y  : DASS_D
    X  : Stress
    M  : FAH

Covariates:
 IB_neg

Sample
Size:  228

Custom
Seed:     123456

**************************************************************************
OUTCOME VARIABLE:
 FAH

Model Summary
          R       R-sq        MSE     F(HC3)        df1        df2          p
      ,4453      ,1983    71,9941    33,0793     2,0000   225,0000      ,0000

Model
              coeff    se(HC3)          t          p       LLCI       ULCI
constant     1,1193     2,3432      ,4777      ,6333    -3,4980     5,7367
Stress        ,0606      ,0253     2,3914      ,0176      ,0107      ,1105
IB_neg        ,9294      ,1208     7,6966      ,0000      ,6914     1,1673

Standardized coefficients
            coeff
Stress      ,1515
IB_neg      ,4246

**************************************************************************
OUTCOME VARIABLE:
 DASS_D

Model Summary
          R       R-sq        MSE     F(HC3)        df1        df2          p
      ,6699      ,4487     9,7934    66,1692     2,0000   225,0000      ,0000

Model
              coeff    se(HC3)          t          p       LLCI       ULCI
constant    -1,3579      ,6316    -2,1499      ,0326    -2,6025     -,1133
Stress        ,0028      ,0101      ,2813      ,7787     -,0171      ,0228
FAH           ,2969      ,0262    11,3170      ,0000      ,2452      ,3486

Standardized coefficients
            coeff
Stress      ,0160
FAH         ,6675


************** TOTAL, DIRECT, AND INDIRECT EFFECTS OF X ON Y **************

Direct effect of X on Y
     Effect    se(HC3)          t          p       LLCI       ULCI      c'_cs
      ,0028      ,0101      ,2813      ,7787     -,0171      ,0228      ,0160

Indirect effect(s) of X on Y:
        Effect     BootSE   BootLLCI   BootULCI
FAH      ,0180      ,0074      ,0035      ,0328
```

```
Completely standardized indirect effect(s) of X on Y:
        Effect     BootSE   BootLLCI   BootULCI
FAH      ,1011      ,0412      ,0202      ,1808

Total effect of X on Y (sum of direct and indirect effects)
     Effect     BootSE   BootLLCI   BootULCI
      ,0208      ,0122     -,0025      ,0447

*********** BOOTSTRAP RESULTS FOR REGRESSION MODEL PARAMETERS ************

OUTCOME VARIABLE:
 FAH

             Coeff   BootMean     BootSE   BootLLCI   BootULCI
constant    1,1193     1,1303     2,3504    -3,4632     5,8480
Stress       ,0606      ,0606      ,0248      ,0121      ,1097
IB_neg       ,9294      ,9288      ,1203      ,6881     1,1617

----------

OUTCOME VARIABLE:
 DASS_D

             Coeff   BootMean     BootSE   BootLLCI   BootULCI
constant   -1,3579    -1,3721      ,6199    -2,5705     -,1416
Stress       ,0028      ,0030      ,0100     -,0169      ,0219
FAH          ,2969      ,2972      ,0259      ,2457      ,3480

*********************** ANALYSIS NOTES AND ERRORS ************************

Level of confidence for all confidence intervals in output:
  95,0000

Number of bootstrap samples for percentile bootstrap confidence intervals:
  10000

NOTE: A heteroscedasticity consistent standard error and covariance matrix
estimator was used.

NOTE: Total effect model generated only when all covariates are specified in all
      models of M and Y.
```

Anxiety outcomes (model 4)

```
Model  : 4
    Y  : DASS_A
    X  : Stress
    M  : FAH

Covariates:
 IB_neg

Sample
Size:  228

Custom
Seed:     123456

**************************************************************************
OUTCOME VARIABLE:
 FAH

Model Summary
          R       R-sq        MSE     F(HC3)        df1        df2          p
      ,4453      ,1983    71,9941    33,0793     2,0000   225,0000      ,0000
```

Model

	coeff	se(HC3)	t	p	LLCI	ULCI
constant	1,1193	2,3432	,4777	,6333	-3,4980	5,7367
Stress	,0606	,0253	2,3914	,0176	,0107	,1105
IB_neg	,9294	,1208	7,6966	,0000	,6914	1,1673

Standardized coefficients

	coeff
Stress	,1515
IB_neg	,4246

****
OUTCOME VARIABLE:
DASS_A

Model Summary

R	R-sq	MSE	F(HC3)	df1	df2	p
,6563	,4307	7,4549	86,5133	2,0000	225,0000	,0000

Model

	coeff	se(HC3)	t	p	LLCI	ULCI
constant	-2,1459	,4329	-4,9565	,0000	-2,9990	-1,2927
Stress	,0232	,0076	3,0640	,0025	,0083	,0381
FAH	,2361	,0201	11,7499	,0000	,1965	,2757

Standardized coefficients

	coeff
Stress	,1517
FAH	,6183

************** TOTAL, DIRECT, AND INDIRECT EFFECTS OF X ON Y **************

Direct effect of X on Y

Effect	se(HC3)	t	p	LLCI	ULCI	c'_cs
,0232	,0076	3,0640	,0025	,0083	,0381	,1517

Indirect effect(s) of X on Y:

	Effect	BootSE	BootLLCI	BootULCI
FAH	,0143	,0060	,0028	,0263

Completely standardized indirect effect(s) of X on Y:

	Effect	BootSE	BootLLCI	BootULCI
FAH	,0936	,0381	,0187	,1687

Total effect of X on Y (sum of direct and indirect effects)

Effect	BootSE	BootLLCI	BootULCI
,0375	,0092	,0193	,0553

*********** BOOTSTRAP RESULTS FOR REGRESSION MODEL PARAMETERS ************

OUTCOME VARIABLE:
FAH

	Coeff	BootMean	BootSE	BootLLCI	BootULCI
constant	1,1193	1,1303	2,3504	-3,4632	5,8480
Stress	,0606	,0606	,0248	,0121	,1097
IB_neg	,9294	,9288	,1203	,6881	1,1617

OUTCOME VARIABLE:
DASS_A

	Coeff	BootMean	BootSE	BootLLCI	BootULCI
constant	-2,1459	-2,1484	,4258	-2,9749	-1,3031
Stress	,0232	,0232	,0075	,0087	,0380
FAH	,2361	,2362	,0198	,1968	,2747

```
********************** ANALYSIS NOTES AND ERRORS ************************

Level of confidence for all confidence intervals in output:
  95,0000

Number of bootstrap samples for percentile bootstrap confidence intervals:
  10000

NOTE: A heteroscedasticity consistent standard error and covariance matrix
estimator was used.

NOTE: Total effect model generated only when all covariates are specified in all
      models of M and Y.
```

Stress-related outcomes (model 4)

```
Model  : 4
    Y  : DASS_S
    X  : Stress
    M  : FAH

Covariates:
 IB_neg

Sample
Size:  228

Custom
Seed:     123456

**************************************************************************
OUTCOME VARIABLE:
 FAH
```

Model Summary

R	R-sq	MSE	F(HC3)	df1	df2	p
,4453	,1983	71,9941	33,0793	2,0000	225,0000	,0000

Model

	coeff	se(HC3)	t	p	LLCI	ULCI
constant	1,1193	2,3432	,4777	,6333	-3,4980	5,7367
Stress	,0606	,0253	2,3914	,0176	,0107	,1105
IB_neg	,9294	,1208	7,6966	,0000	,6914	1,1673

Standardized coefficients

	coeff
Stress	,1515
IB_neg	,4246

```
**************************************************************************
OUTCOME VARIABLE:
 DASS_S
```

Model Summary

R	R-sq	MSE	F(HC3)	df1	df2	p
,6739	,4542	9,0907	98,2814	2,0000	225,0000	,0000

Model

	coeff	se(HC3)	t	p	LLCI	ULCI
constant	-,6199	,5358	-1,1570	,2485	-1,6758	,4359
Stress	,0329	,0097	3,4058	,0008	,0139	,0519
FAH	,2674	,0225	11,8997	,0000	,2231	,3117

Standardized coefficients

	coeff
Stress	,1910
FAH	,6210

```
************** TOTAL, DIRECT, AND INDIRECT EFFECTS OF X ON Y **************

Direct effect of X on Y
     Effect    se(HC3)          t          p       LLCI       ULCI      c'_cs
      ,0329      ,0097     3,4058      ,0008      ,0139      ,0519      ,1910

Indirect effect(s) of X on Y:
        Effect     BootSE   BootLLCI   BootULCI
FAH      ,0162      ,0068      ,0032      ,0298

Completely standardized indirect effect(s) of X on Y:
        Effect     BootSE   BootLLCI   BootULCI
FAH      ,0940      ,0385      ,0185      ,1696

Total effect of X on Y (sum of direct and indirect effects)
     Effect     BootSE   BootLLCI   BootULCI
      ,0491      ,0109      ,0276      ,0703

*********** BOOTSTRAP RESULTS FOR REGRESSION MODEL PARAMETERS ************

OUTCOME VARIABLE:
 FAH

              Coeff   BootMean     BootSE   BootLLCI   BootULCI
constant     1,1193     1,1303     2,3504    -3,4632     5,8480
Stress        ,0606      ,0606      ,0248      ,0121      ,1097
IB_neg        ,9294      ,9288      ,1203      ,6881     1,1617

----------

OUTCOME VARIABLE:
 DASS_S

              Coeff   BootMean     BootSE   BootLLCI   BootULCI
constant     -,6199     -,6247      ,5338    -1,6650      ,4225
Stress        ,0329      ,0329      ,0095      ,0145      ,0515
FAH           ,2674      ,2677      ,0223      ,2237      ,3115

*********************** ANALYSIS NOTES AND ERRORS ************************

Level of confidence for all confidence intervals in output:
  95,0000

Number of bootstrap samples for percentile bootstrap confidence intervals:
  10000

NOTE: A heteroscedasticity consistent standard error and covariance matrix
estimator was used.

NOTE: Total effect model generated only when all covariates are specified in all
      models of M and Y.
```

Positive mental health outcomes (model 14)

```
Model  : 14
    Y  : PMH
    X  : Stress
    M  : FAH
    W  : IB_neg

Covariates:
 IB_pos

Sample
Size:  228

Custom
```

Seed: 123456

****
OUTCOME VARIABLE:
FAH

Model Summary

R	R-sq	MSE	F(HC3)	df1	df2	p
,1352	,0183	87,7662	3,7592	1,0000	226,0000	,0538

Model

	coeff	se(HC3)	t	p	LLCI	ULCI
constant	-2,6802	1,4755	-1,8165	,0706	-5,5877	,2272
Stress	,0541	,0279	1,9389	,0538	-,0009	,1091

****
OUTCOME VARIABLE:
PMH

Model Summary

R	R-sq	MSE	F(HC3)	df1	df2	p
,7557	,5711	11,4044	48,6456	5,0000	222,0000	,0000

Model

	coeff	se(HC3)	t	p	LLCI	ULCI
constant	13,8386	2,0765	6,6645	,0000	9,7466	17,9307
Stress	-,0168	,0117	-1,4372	,1521	-,0398	,0062
FAH	-,3717	,0398	-9,3489	,0000	-,4500	-,2933
IB_neg	,0718	,0656	1,0952	,2746	-,0574	,2011
Int_1	,0125	,0069	1,8099	,0717	-,0011	,0261
IB_pos	,2657	,0946	2,8092	,0054	,0793	,4521

Product terms key:
Int_1 : FAH x IB_neg

Test(s) of highest order unconditional interaction(s):

	R2-chng	F(HC3)	df1	df2	p
M*W	,0099	3,2756	1,0000	222,0000	,0717

Focal predict: FAH (M)
Mod var: IB_neg (W)

Conditional effects of the focal predictor at values of the moderator(s):

IB_neg	Effect	se(HC3)	t	p	LLCI	ULCI
-4,3097	-,4256	,0541	-7,8661	,0000	-,5322	-,3190
,0000	-,3717	,0398	-9,3489	,0000	-,4500	-,2933
4,3097	-,3177	,0448	-7,0887	,0000	-,4061	-,2294

There are no statistical significance transition points within the observed range of the moderator found using the Johnson-Neyman method.

Conditional effect of focal predictor at values of the moderator:

IB_neg	Effect	se(HC3)	t	p	LLCI	ULCI
-11,3026	-,5131	,0943	-5,4416	,0000	-,6989	-,3273
-10,2074	-,4994	,0875	-5,7104	,0000	-,6717	-,3270
-9,1122	-,4857	,0807	-6,0150	,0000	-,6448	-,3266
-8,0169	-,4720	,0742	-6,3604	,0000	-,6182	-,3257
-6,9217	-,4583	,0679	-6,7512	,0000	-,5920	-,3245
-5,8264	-,4446	,0618	-7,1895	,0000	-,5664	-,3227
-4,7312	-,4309	,0562	-7,6716	,0000	-,5415	-,3202
-3,6360	-,4172	,0510	-8,1817	,0000	-,5176	-,3167
-2,5407	-,4035	,0465	-8,6817	,0000	-,4950	-,3119
-1,4455	-,3898	,0428	-9,1002	,0000	-,4742	-,3053
-,3503	-,3760	,0403	-9,3325	,0000	-,4555	-,2966
,7450	-,3623	,0391	-9,2707	,0000	-,4394	-,2853
1,8402	-,3486	,0393	-8,8660	,0000	-,4261	-,2711
2,9355	-,3349	,0410	-8,1724	,0000	-,4157	-,2542
4,0307	-,3212	,0439	-7,3165	,0000	-,4078	-,2347
5,1259	-,3075	,0479	-6,4259	,0000	-,4018	-,2132

```
     6,2212     -,2938      ,0526    -5,5850      ,0000     -,3975     -,1901
     7,3164     -,2801      ,0580    -4,8327      ,0000     -,3943     -,1659
     8,4117     -,2664      ,0638    -4,1778      ,0000     -,3921     -,1407
     9,5069     -,2527      ,0699    -3,6146      ,0004     -,3905     -,1149
    10,6021     -,2390      ,0763    -3,1318      ,0020     -,3894     -,0886
    11,6974     -,2253      ,0829    -2,7173      ,0071     -,3887     -,0619

Data for visualizing the conditional effect of the focal predictor:
Paste text below into a SPSS syntax window and execute to produce plot.

DATA LIST FREE/
   FAH        IB_neg     PMH        .
BEGIN DATA.
    -9,4344    -4,3097    21,9453
      ,0000    -4,3097    17,9301
     9,4344    -4,3097    13,9149
    -9,4344      ,0000    21,7461
      ,0000      ,0000    18,2396
     9,4344      ,0000    14,7332
    -9,4344     4,3097    21,5468
      ,0000     4,3097    18,5492
     9,4344     4,3097    15,5515
END DATA.
GRAPH/SCATTERPLOT=
 FAH      WITH     PMH      BY       IB_neg   .


****************** DIRECT AND INDIRECT EFFECTS OF X ON Y *****************

Direct effect of X on Y
     Effect    se(HC3)          t          p       LLCI       ULCI
     -,0168      ,0117    -1,4372      ,1521     -,0398      ,0062

Conditional indirect effects of X on Y:

INDIRECT EFFECT:
 Stress      ->    FAH         ->    PMH

     IB_neg     Effect     BootSE   BootLLCI   BootULCI
    -4,3097     -,0230      ,0115     -,0451     -,0001
      ,0000     -,0201      ,0100     -,0396     -,0001
     4,3097     -,0172      ,0088     -,0349     -,0001

      Index of moderated mediation:
            Index     BootSE   BootLLCI   BootULCI
IB_neg      ,0007      ,0005     -,0001      ,0017

*********** BOOTSTRAP RESULTS FOR REGRESSION MODEL PARAMETERS ************

OUTCOME VARIABLE:
 FAH

              Coeff   BootMean     BootSE   BootLLCI   BootULCI
constant    -2,6802    -2,7046     1,4510    -5,4900      ,1702
Stress        ,0541      ,0545      ,0274      ,0002      ,1076

----------

OUTCOME VARIABLE:
 PMH

              Coeff   BootMean     BootSE   BootLLCI   BootULCI
constant    13,8386    13,9289     1,9322    10,0398    17,6774
Stress       -,0168     -,0170      ,0112     -,0383      ,0051
FAH          -,3717     -,3723      ,0374     -,4423     -,2965
IB_neg        ,0718      ,0662      ,0642     -,0636      ,1894
Int_1         ,0125      ,0119      ,0065     -,0011      ,0245
IB_pos        ,2657      ,2621      ,0882      ,0934      ,4407

*********************** ANALYSIS NOTES AND ERRORS ************************
```

Level of confidence for all confidence intervals in output:
  95,0000

Number of bootstrap samples for percentile bootstrap confidence intervals:
  10000

W values in conditional tables are the mean and +/- SD from the mean.

NOTE: A heteroscedasticity consistent standard error and covariance matrix estimator was used.

NOTE: The following variables were mean centered prior to analysis:
IB_neg FAH

NOTE: Standardized coefficients are not available for models with moderators.

Positive mental health outcomes (model 4)

```
Model  : 4
    Y  : PMH
    X  : Stress
    M  : FAH

Covariates:
 IB_neg   IB_pos

Sample
Size:  228

Custom
Seed:     123456

**************************************************************************
OUTCOME VARIABLE:
 FAH
```

Model Summary

R	R-sq	MSE	F(HC3)	df1	df2	p
,4453	,1983	71,9941	33,0793	2,0000	225,0000	,0000

Model

	coeff	se(HC3)	t	p	LLCI	ULCI
constant	1,1193	2,3432	,4777	,6333	-3,4980	5,7367
Stress	,0606	,0253	2,3914	,0176	,0107	,1105
IB_neg	,9294	,1208	7,6966	,0000	,6914	1,1673

Standardized coefficients

	coeff
Stress	,1515
IB_neg	,4246

```
**************************************************************************
OUTCOME VARIABLE:
 PMH
```

Model Summary

R	R-sq	MSE	F(HC3)	df1	df2	p
,7480	,5596	11,6053	66,2800	3,0000	224,0000	,0000

Model

	coeff	se(HC3)	t	p	LLCI	ULCI
constant	21,8038	2,4524	8,8908	,0000	16,9710	26,6365
Stress	-,0158	,0115	-1,3785	,1694	-,0385	,0068
FAH	-,3528	,0386	-9,1422	,0000	-,4288	-,2767
IB_pos	,2315	,0888	2,6062	,0098	,0565	,4066

Standardized coefficients
coeff

```
Stress     -,0733
FAH        -,6527
IB_pos      ,1804


************** TOTAL, DIRECT, AND INDIRECT EFFECTS OF X ON Y **************

Direct effect of X on Y
     Effect    se(HC3)          t          p       LLCI       ULCI      c'_cs
     -,0158      ,0115    -1,3785      ,1694     -,0385      ,0068     -,0733

Indirect effect(s) of X on Y:
        Effect     BootSE   BootLLCI   BootULCI
FAH     -,0214      ,0085     -,0380     -,0044

Completely standardized indirect effect(s) of X on Y:
        Effect     BootSE   BootLLCI   BootULCI
FAH     -,0988      ,0392     -,1748     -,0207

Total effect of X on Y (sum of direct and indirect effects)
     Effect     BootSE   BootLLCI   BootULCI
     -,0372      ,0137     -,0643     -,0103

*********** BOOTSTRAP RESULTS FOR REGRESSION MODEL PARAMETERS ************

OUTCOME VARIABLE:
 FAH

             Coeff   BootMean     BootSE   BootLLCI   BootULCI
constant    1,1193     1,1303     2,3504    -3,4632     5,8480
Stress       ,0606      ,0606      ,0248      ,0121      ,1097
IB_neg       ,9294      ,9288      ,1203      ,6881     1,1617

----------

OUTCOME VARIABLE:
 PMH

             Coeff   BootMean     BootSE   BootLLCI   BootULCI
constant   21,8038    21,8935     2,2797    17,3556    26,3043
Stress      -,0158     -,0162      ,0112     -,0379      ,0057
FAH         -,3528     -,3547      ,0365     -,4245     -,2805
IB_pos       ,2315      ,2297      ,0832      ,0673      ,3959

********************** ANALYSIS NOTES AND ERRORS ************************

Level of confidence for all confidence intervals in output:
  95,0000

Number of bootstrap samples for percentile bootstrap confidence intervals:
  10000

NOTE: A heteroscedasticity consistent standard error and covariance matrix
estimator was used.

NOTE: Total effect model generated only when all covariates are specified in all
      models of M and Y.
```

A4b: High stress condition

Depression outcomes (model 14)

```
Model  : 14
    Y  : DASS_D_p
    X  : Stress_p
    M  : FAH_p
    W  : IB_neg
```

```
Covariates:
 IB_neg

Sample
Size:  228

Custom
Seed:     123456

**************************************************************************
OUTCOME VARIABLE:
 FAH_p

Model Summary
          R       R-sq        MSE     F(HC3)        df1        df2          p
      ,4480      ,2007    61,4255    25,5062     2,0000   225,0000      ,0000

Model
              coeff    se(HC3)          t          p       LLCI       ULCI
constant    -4,8438     1,7256    -2,8070      ,0054    -8,2443    -1,4433
Stress_p      ,0815      ,0279     2,9269      ,0038      ,0266      ,1364
IB_neg        ,7597      ,1255     6,0521      ,0000      ,5123     1,0070

**************************************************************************
OUTCOME VARIABLE:
 DASS_D_p

Model Summary
          R       R-sq        MSE     F(HC3)        df1        df2          p
      ,7183      ,5160     8,4596    55,9560     4,0000   223,0000      ,0000

Model
              coeff    se(HC3)          t          p       LLCI       ULCI
constant     4,4805      ,6833     6,5571      ,0000     3,1340     5,8271
Stress_p      ,0059      ,0107      ,5547      ,5797     -,0152      ,0271
FAH_p         ,2786      ,0276    10,0898      ,0000      ,2242      ,3330
IB_neg        ,1583      ,0494     3,2061      ,0015      ,0610      ,2557
Int_1         ,0157      ,0055     2,8363      ,0050      ,0048      ,0266

Product terms key:
 Int_1    :        FAH_p    x        IB_neg

Test(s) of highest order unconditional interaction(s):
       R2-chng     F(HC3)        df1        df2          p
M*W      ,0204     8,0448     1,0000   223,0000      ,0050
----------
    Focal predict: FAH_p    (M)
          Mod var: IB_neg   (W)

Conditional effects of the focal predictor at values of the moderator(s):

     IB_neg     Effect    se(HC3)          t          p       LLCI       ULCI
    -4,3097      ,2111      ,0430     4,9090      ,0000      ,1263      ,2958
      ,0000      ,2786      ,0276    10,0898      ,0000      ,2242      ,3330
     4,3097      ,3461      ,0285    12,1646      ,0000      ,2900      ,4022

Moderator value(s) defining Johnson-Neyman significance region(s):
      Value    % below    % above
    -9,3137      ,8772    99,1228

Conditional effect of focal predictor at values of the moderator:
     IB_neg     Effect    se(HC3)          t          p       LLCI       ULCI
   -11,3026      ,1015      ,0776     1,3079      ,1923     -,0514      ,2544
   -10,1526      ,1195      ,0716     1,6685      ,0966     -,0216      ,2607
    -9,3137      ,1327      ,0673     1,9707      ,0500      ,0000      ,2653
    -9,0026      ,1375      ,0657     2,0924      ,0375      ,0080      ,2671
    -7,8526      ,1556      ,0599     2,5960      ,0101      ,0375      ,2736
    -6,7026      ,1736      ,0542     3,2005      ,0016      ,0667      ,2804
    -5,5526      ,1916      ,0487     3,9334      ,0001      ,0956      ,2876
    -4,4026      ,2096      ,0434     4,8284      ,0000      ,1241      ,2952
```

-3,2526	,2276	,0384	5,9223	,0000	,1519	,3034
-2,1026	,2456	,0339	7,2413	,0000	,1788	,3125
-,9526	,2637	,0301	8,7649	,0000	,2044	,3229
,1974	,2817	,0272	10,3565	,0000	,2281	,3353
1,3474	,2997	,0256	11,7073	,0000	,2493	,3501
2,4974	,3177	,0255	12,4466	,0000	,2674	,3680
3,6474	,3357	,0270	12,4382	,0000	,2825	,3889
4,7974	,3538	,0298	11,8827	,0000	,2951	,4124
5,9474	,3718	,0335	11,0857	,0000	,3057	,4379
7,0974	,3898	,0380	10,2584	,0000	,3149	,4647
8,2474	,4078	,0429	9,4978	,0000	,3232	,4924
9,3974	,4258	,0482	8,8329	,0000	,3308	,5208
10,5474	,4438	,0537	8,2629	,0000	,3380	,5497
11,6974	,4619	,0594	7,7766	,0000	,3448	,5789

Data for visualizing the conditional effect of the focal predictor:
Paste text below into a SPSS syntax window and execute to produce plot.

```
DATA LIST FREE/
   FAH_p      IB_neg     DASS_D_p   .
BEGIN DATA.
    -8,7277    -4,3097     2,3093
      ,0000    -4,3097     4,1514
     8,7277    -4,3097     5,9936
    -8,7277      ,0000     2,4024
      ,0000      ,0000     4,8338
     8,7277      ,0000     7,2653
    -8,7277     4,3097     2,4955
      ,0000     4,3097     5,5162
     8,7277     4,3097     8,5370
END DATA.
GRAPH/SCATTERPLOT=
 FAH_p    WITH     DASS_D_p BY       IB_neg   .
```

****************** DIRECT AND INDIRECT EFFECTS OF X ON Y *****************

Direct effect of X on Y

Effect	se(HC3)	t	p	LLCI	ULCI
,0059	,0107	,5547	,5797	-,0152	,0271

Conditional indirect effects of X on Y:

INDIRECT EFFECT:
Stress_p -> FAH_p -> DASS_D_p

IB_neg	Effect	BootSE	BootLLCI	BootULCI
-4,3097	,0172	,0065	,0056	,0312
,0000	,0227	,0079	,0078	,0389
4,3097	,0282	,0098	,0096	,0488

Index of moderated mediation:

	Index	BootSE	BootLLCI	BootULCI
IB_neg	,0013	,0007	,0003	,0028

*********** BOOTSTRAP RESULTS FOR REGRESSION MODEL PARAMETERS ************

OUTCOME VARIABLE:
FAH_p

	Coeff	BootMean	BootSE	BootLLCI	BootULCI
constant	-4,8438	-4,8966	1,6974	-8,2316	-1,5421
Stress_p	,0815	,0824	,0275	,0286	,1374
IB_neg	,7597	,7593	,1248	,5095	,9978

OUTCOME VARIABLE:
DASS_D_p

	Coeff	BootMean	BootSE	BootLLCI	BootULCI
constant	4,4805	4,4746	,6639	3,1487	5,7766
Stress_p	,0059	,0060	,0105	-,0146	,0268
FAH_p	,2786	,2786	,0264	,2258	,3303
IB_neg	,1583	,1571	,0476	,0610	,2478
Int_1	,0157	,0157	,0053	,0054	,0259

********************** ANALYSIS NOTES AND ERRORS ************************

Level of confidence for all confidence intervals in output:
95,0000

Number of bootstrap samples for percentile bootstrap confidence intervals:
10000

W values in conditional tables are the mean and +/- SD from the mean.

NOTE: A heteroscedasticity consistent standard error and covariance matrix estimator was used.

NOTE: The following variables were mean centered prior to analysis:
IB_neg FAH_p

NOTE: Standardized coefficients are not available for models with moderators.

Anxiety outcomes (model 4)

Model : 4
Y : DASS_A_p
X : Stress_p
M : FAH_p

Covariates:
IB_neg

Sample
Size: 228

Custom
Seed: 123456

****
OUTCOME VARIABLE:
FAH_p

Model Summary

R	R-sq	MSE	F(HC3)	df1	df2	p
,4480	,2007	61,4255	25,5062	2,0000	225,0000	,0000

Model

	coeff	se(HC3)	t	p	LLCI	ULCI
constant	2,6420	2,4826	1,0642	,2884	-2,2500	7,5341
Stress_p	,0815	,0279	2,9269	,0038	,0266	,1364
IB_neg	,7597	,1255	6,0521	,0000	,5123	1,0070

Standardized coefficients

	coeff
Stress_p	,1978
IB_neg	,3751

****
OUTCOME VARIABLE:
DASS_A_p

Model Summary

R	R-sq	MSE	F(HC3)	df1	df2	p
,6270	,3932	7,2948	77,9602	2,0000	225,0000	,0000

Model

	coeff	se(HC3)	t	p	LLCI	ULCI

```
constant    -1,6335      ,5545    -2,9457      ,0036    -2,7263     -,5407
Stress_p      ,0053      ,0088      ,6062      ,5450     -,0119      ,0226
FAH_p         ,2445      ,0204    12,0055      ,0000      ,2043      ,2846

Standardized coefficients
              coeff
Stress_p      ,0326
FAH_p         ,6181


************** TOTAL, DIRECT, AND INDIRECT EFFECTS OF X ON Y **************

Direct effect of X on Y
     Effect    se(HC3)          t          p       LLCI       ULCI      c'_cs
      ,0053      ,0088      ,6062      ,5450     -,0119      ,0226      ,0326

Indirect effect(s) of X on Y:
          Effect     BootSE   BootLLCI   BootULCI
FAH_p      ,0199      ,0068      ,0070      ,0338

Completely standardized indirect effect(s) of X on Y:
          Effect     BootSE   BootLLCI   BootULCI
FAH_p      ,1222      ,0398      ,0435      ,2008

Total effect of X on Y (sum of direct and indirect effects)
     Effect     BootSE   BootLLCI   BootULCI
      ,0252      ,0108      ,0048      ,0475

*********** BOOTSTRAP RESULTS FOR REGRESSION MODEL PARAMETERS ************

OUTCOME VARIABLE:
 FAH_p

              Coeff   BootMean     BootSE   BootLLCI   BootULCI
constant     2,6420     2,5953     2,4746    -2,2005     7,4600
Stress_p      ,0815      ,0824      ,0275      ,0286      ,1374
IB_neg        ,7597      ,7593      ,1248      ,5095      ,9978

----------

OUTCOME VARIABLE:
 DASS_A_p

              Coeff   BootMean     BootSE   BootLLCI   BootULCI
constant    -1,6335    -1,6415      ,5468    -2,7232     -,5640
Stress_p      ,0053      ,0055      ,0087     -,0114      ,0231
FAH_p         ,2445      ,2442      ,0201      ,2046      ,2837

*********************** ANALYSIS NOTES AND ERRORS ************************

Level of confidence for all confidence intervals in output:
  95,0000

Number of bootstrap samples for percentile bootstrap confidence intervals:
  10000

NOTE: A heteroscedasticity consistent standard error and covariance matrix
estimator was used.

NOTE: Total effect model generated only when all covariates are specified in all
      models of M and Y.
```

Stress-related outcomes (model 4)

```
Model  : 4
    Y  : DASS_S_p
    X  : Stress_p
    M  : FAH_p
```

Covariates:
IB_neg

Sample
Size: 228

Custom
Seed: 123456

****
OUTCOME VARIABLE:
FAH_p

Model Summary

R	R-sq	MSE	F(HC3)	df1	df2	p
,4480	,2007	61,4255	25,5062	2,0000	225,0000	,0000

Model

	coeff	se(HC3)	t	p	LLCI	ULCI
constant	2,6420	2,4826	1,0642	,2884	-2,2500	7,5341
Stress_p	,0815	,0279	2,9269	,0038	,0266	,1364
IB_neg	,7597	,1255	6,0521	,0000	,5123	1,0070

Standardized coefficients

	coeff
Stress_p	,1978
IB_neg	,3751

****
OUTCOME VARIABLE:
DASS_S_p

Model Summary

R	R-sq	MSE	F(HC3)	df1	df2	p
,6955	,4837	9,2105	89,7599	3,0000	224,0000	,0000

Model

	coeff	se(HC3)	t	p	LLCI	ULCI
constant	-2,8348	,8487	-3,3400	,0010	-4,5073	-1,1623
Stress_p	,0261	,0099	2,6321	,0091	,0066	,0456
FAH_p	,2780	,0280	9,9364	,0000	,2229	,3332
IB_neg	,1409	,0525	2,6815	,0079	,0373	,2444

Standardized coefficients

	coeff
Stress_p	,1317
FAH_p	,5783
IB_neg	,1447

************************** TOTAL EFFECT MODEL ****************************
OUTCOME VARIABLE:
DASS_S_p

Model Summary

R	R-sq	MSE	F(HC3)	df1	df2	p
,4652	,2164	13,9183	30,9078	2,0000	225,0000	,0000

Model

	coeff	se(HC3)	t	p	LLCI	ULCI
constant	-2,1002	1,1156	-1,8826	,0611	-4,2985	,0982
Stress_p	,0488	,0124	3,9227	,0001	,0243	,0733
IB_neg	,3521	,0552	6,3825	,0000	,2434	,4608

Standardized coefficients

	coeff
Stress_p	,2461
IB_neg	,3616

************** TOTAL, DIRECT, AND INDIRECT EFFECTS OF X ON Y **************

```
Total effect of X on Y
     Effect    se(HC3)          t          p       LLCI       ULCI       c_cs
      ,0488      ,0124     3,9227      ,0001      ,0243      ,0733      ,2461

Direct effect of X on Y
     Effect    se(HC3)          t          p       LLCI       ULCI      c'_cs
      ,0261      ,0099     2,6321      ,0091      ,0066      ,0456      ,1317

Indirect effect(s) of X on Y:
          Effect     BootSE   BootLLCI   BootULCI
FAH_p      ,0227      ,0079      ,0080      ,0389

Completely standardized indirect effect(s) of X on Y:
          Effect     BootSE   BootLLCI   BootULCI
FAH_p      ,1144      ,0378      ,0412      ,1893

*********** BOOTSTRAP RESULTS FOR REGRESSION MODEL PARAMETERS ************

OUTCOME VARIABLE:
 FAH_p

              Coeff   BootMean     BootSE   BootLLCI   BootULCI
constant     2,6420     2,5953     2,4746    -2,2005     7,4600
Stress_p      ,0815      ,0824      ,0275      ,0286      ,1374
IB_neg        ,7597      ,7593      ,1248      ,5095      ,9978

----------

OUTCOME VARIABLE:
 DASS_S_p

              Coeff   BootMean     BootSE   BootLLCI   BootULCI
constant    -2,8348    -2,8404      ,8363    -4,4706    -1,1907
Stress_p      ,0261      ,0263      ,0098      ,0071      ,0455
FAH_p         ,2780      ,2782      ,0273      ,2237      ,3310
IB_neg        ,1409      ,1406      ,0516      ,0393      ,2410

********************** ANALYSIS NOTES AND ERRORS ************************

Level of confidence for all confidence intervals in output:
  95,0000

Number of bootstrap samples for percentile bootstrap confidence intervals:
  10000

NOTE: A heteroscedasticity consistent standard error and covariance matrix
estimator was used.
```

Positive mental health outcomes (model 4)

```
Model  : 4
    Y  : PMH_p
    X  : Stress_p
    M  : FAH_p

Covariates:
 IB_neg   IB_pos

Sample
Size:  228

Custom
Seed:     123456

**************************************************************************
OUTCOME VARIABLE:
 FAH_p
```

Model Summary

R	R-sq	MSE	F(HC3)	df1	df2	p
,4480	,2007	61,4255	25,5062	2,0000	225,0000	,0000

Model

	coeff	se(HC3)	t	p	LLCI	ULCI
constant	2,6420	2,4826	1,0642	,2884	-2,2500	7,5341
Stress_p	,0815	,0279	2,9269	,0038	,0266	,1364
IB_neg	,7597	,1255	6,0521	,0000	,5123	1,0070

Standardized coefficients

	coeff
Stress_p	,1978
IB_neg	,3751

****
OUTCOME VARIABLE:
PMH_p

Model Summary

R	R-sq	MSE	F(HC3)	df1	df2	p
,6758	,4568	14,3342	60,4434	3,0000	224,0000	,0000

Model

	coeff	se(HC3)	t	p	LLCI	ULCI
constant	22,9732	1,9620	11,7092	,0000	19,1069	26,8394
Stress_p	-,0096	,0127	-,7570	,4498	-,0348	,0155
FAH_p	-,3554	,0385	-9,2419	,0000	-,4312	-,2796
IB_pos	,1672	,0732	2,2830	,0234	,0229	,3115

Standardized coefficients

	coeff
Stress_p	-,0400
FAH_p	-,6079
IB_pos	,1302

************** TOTAL, DIRECT, AND INDIRECT EFFECTS OF X ON Y **************

Direct effect of X on Y

Effect	se(HC3)	t	p	LLCI	ULCI	c'_cs
-,0096	,0127	-,7570	,4498	-,0348	,0155	-,0400

Indirect effect(s) of X on Y:

	Effect	BootSE	BootLLCI	BootULCI
FAH_p	-,0290	,0102	-,0501	-,0101

Completely standardized indirect effect(s) of X on Y:

	Effect	BootSE	BootLLCI	BootULCI
FAH_p	-,1202	,0404	-,2025	-,0425

Total effect of X on Y (sum of direct and indirect effects)

Effect	BootSE	BootLLCI	BootULCI
-,0386	,0166	-,0723	-,0066

*********** BOOTSTRAP RESULTS FOR REGRESSION MODEL PARAMETERS ************

OUTCOME VARIABLE:
FAH_p

	Coeff	BootMean	BootSE	BootLLCI	BootULCI
constant	2,6420	2,5953	2,4746	-2,2005	7,4600
Stress_p	,0815	,0824	,0275	,0286	,1374
IB_neg	,7597	,7593	,1248	,5095	,9978

OUTCOME VARIABLE:
PMH_p

	Coeff	BootMean	BootSE	BootLLCI	BootULCI
constant	22,9732	22,9898	1,8935	19,2198	26,6680
Stress_p	-,0096	-,0102	,0124	-,0351	,0139
FAH_p	-,3554	-,3554	,0373	-,4277	-,2815
IB_pos	,1672	,1678	,0707	,0312	,3092

********************** ANALYSIS NOTES AND ERRORS ************************

Level of confidence for all confidence intervals in output:
95,0000

Number of bootstrap samples for percentile bootstrap confidence intervals:
10000

NOTE: A heteroscedasticity consistent standard error and covariance matrix estimator was used.

NOTE: Total effect model generated only when all covariates are specified in all models of M and Y.
